# Intracellular and nuclear CXCR4 signaling promotes terminal erythroblast differentiation and enucleation

**DOI:** 10.1126/scisignal.adt2678

**Published:** 2025-06-17

**Authors:** Julia Christine Gutjahr, Elin Hub, Caroline Amy Anderson, Maryna Samus, Katharina Artinger, Esteban A. Gomez, Christoph Ratswohl, Natalie Wickli, Mandy Raum, Neil Dufton, Jesmond Dalli, Jemima Joanne Burden, Johan Duchene, Antal Rot

**Affiliations:** 1 Centre for Microvascular Research, William Harvey Research Institute, Faculty of Medicine and Dentistry, Queen Mary University of London, London EC1M 6BQ, UK; 2 Institute of Cell Biology and Immunology Thurgau (BITG), University of Konstanz, Kreuzlingen 8280, Switzerland; 3 Department of Biology, University of Konstanz, Konstanz 78464, Germany; 4 Center for Translational Medicine and Therapeutics, William Harvey Research Institute, Faculty of Medicine and Dentistry, Queen Mary University of London, London EC1M 6BQ, UK; 5 Division of Nephrology, Department of Internal Medicine, Medical University of Graz, Graz 8036, Austria; 6 Biochemical Pharmacology, William Harvey Research Institute, Faculty of Medicine and Dentistry, Queen Mary University of London, London EC1M 6BQ, UK; 7 Laboratory for Molecular Cell Biology, University College London, Gower Street, London, WC1E 6BT, UK; 8 Institute for Cardiovascular Prevention, Ludwig-Maximilians University, Munich 80336, Germany

## Abstract

The chemokine CXCL12 signals through its receptor CXCR4 to stimulate the migration of all leukocyte types and multiple other cell types. Here, we report that CXCR4 is expressed in mouse erythroblasts, the bone marrow erythroid precursors, in which it mediated erythrocyte generation instead of chemotaxis. CXCR4 signaling promoted homeostatic erythroblast maturation and increased the expression of genes mainly involved in metabolism and chromatin organization. Consequently, genetic depletion of CXCR4 in erythroblasts inhibited late erythropoiesis and diminished bone marrow erythroid outputs. Binding of CXCL12 to CXCR4 stimulated its rapid endocytosis and translocation together with Gα_i_ or phosphorylated β-arrestin1 into distinct intracellular compartments, including the nuclear envelope and nucleus. CXCL12 signaling promoted erythroblast elongation and the condensation and excentric positioning of nuclei and stimulated rapid perinuclear Ca^2+^ transients that immediately preceded erythroblast enucleation. These findings highlight previously uncharacterized physiological roles for CXCR4 and bone marrow–derived CXCL12 in erythropoiesis.

## Introduction

Chemokines are structurally homologous molecular signals that ligate their cognate cell surface receptors to induce, most notably, directed cell migration (1). The primeval chemokine CXCL12 binds to CXCR4, its sole classical receptor expressed on the plasma membranes of multiple cells, to stimulate their chemotaxis and other cell responses underlying diverse (patho)physiological functions (2). Genetic ablation of either CXCL12 or CXCR4 is lethal because of developmental defects within the hematopoietic system, the brain, and the heart (3–5). CXCL12 is produced constitutively in the bone marrow (BM) by specialized reticular cells (6, 7), whereas CXCR4 has been described and functionally characterized in detail in hematopoietic stem and progenitor cells and practically every hematopoietic lineage (8), bar, hitherto, erythroid cells.

The initial cellular sources of BM erythropoiesis are erythroid progenitor cells, which are called colony-forming unit–erythroid cells, which divide up to five times to give rise to distinct generations of differentiated erythroid cells (9, 10). The earliest of such erythroid lineage–committed cells is the proerythroblast, which sequentially develops into the basophilic (early), polychromatophilic (intermediate), and orthochromatic (late) erythroblasts (11). Proerythroblasts undergo a series of intracellular changes that accompany their differentiation, including successive reduction in nuclear size and the autolysis of intracellular organelles, including mitochondria, the endoplasmic reticulum, the Golgi apparatus, and ribosomes (9). To form mature erythrocytes, mammalian erythroblasts must undergo enucleation, a special form of asymmetric cell division, which leads to the formation of an anuclear reticulocyte and a nucleus-containing pyrenocyte (12, 13). Before enucleation, polarization of the erythroblast nucleus to one cell side takes place, which is followed by protein sorting to two alternative cell poles containing either the future erythrocyte or surrounding the nucleus to be ejected. The reticulocyte is the ultimate BM erythroid cell that enters the circulation to fully mature and become an erythrocyte (14). Chemokine receptors have not been studied systematically in erythroid cells, except for the atypical receptor ACKR1, which is highly abundant in erythroblasts (15). ACKR1 binds to many inflammatory chemokines, as well as dimeric CXCL12 (16).

Here, we found that CXCR4, the sole classical chemokine receptor for CXCL12, is expressed in primary mouse erythroblasts. In contrast to its established role in mediating chemotaxis in other cell types, CXCR4 did not promote erythroblast migration. Instead, it facilitated their terminal maturation and enucleation. These results uncover a previously unappreciated physiological function of CXCR4 and BM-derived CXCL12 in regulating erythropoiesis.

## Results

### CXCR4 is expressed in erythroblasts

**Fig. 1:**
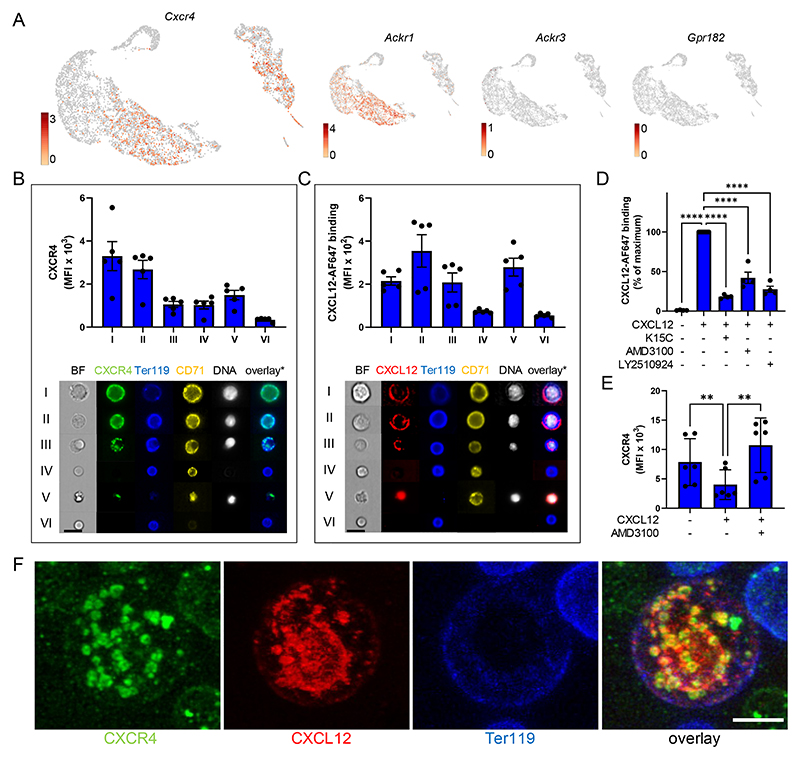
BM erythroid cells express functional CXCR4. **(A)** Relative transcript abundance (log_2_) of *Cxcr4, Ackr1, Ackr3*, and *Gpr182* from the scRNA-seq analysis of murine CD71^+^ BM erythroid cells, displayed as a UMAP. (**B**) Top: Anti-CXCR4 antibody reactivity with murine BM proerythroblasts (I), early erythroblasts (II), late erythroblasts (III), reticulocytes (IV), pyrenocytes (V), and erythrocytes (VI) as measured by flow cytometry. Data are from five mice. Bottom: Imaging flow cytometry of the indicated targets in the indicated cell types. The overlay (*) shows both CXCR4 and Ter119 signals. (**C**) Top: CXCL12-AF647 binding by the indicated erythroid cell subsets, as described in (B), measured in flow cytometry. Data are from five mice. Bottom: Imaging flow cytometry of the indicated targets in the indicated cell types. The overlay (*) shows CXCL12, Ter119, and Hoechst (DNA) signals. (**D**) CXCL12-AF647 binding to CD71^+^Ter119^+^ erythroblasts and its inhibition by an anti–N-terminal CXCL12 antibody K15C and the CXCR4 antagonists AMD3100 and LY2510924. Data are from four mice. ****P < 0.001 by one-way ANOVA with Dunnett’s multiple comparisons test. (**E**) Surface CXCR4 immunoreactivity in CD71^+^Ter119^+^ erythroblasts and the effect of CXCL12 and pretreatment with AMD3100 on binding. Data are from six mice. **P < 0.01 by one-way ANOVA with Dunnett’s multiple comparisons test. (**F**) Confocal microscopy representation of CXCR4 immunoreactivity in a viable erythroblast. Freshly isolated erythroblasts were pre-stained with an anti-Ter119 antibody, labeled with the non-blocking anti-CXCR4 antibody 2B11, and stimulated with CXCL12-AF647. Because the cells remained viable for the duration of the experiment, 2B11 labeled CXCR4 on the plasma membrane only. Therefore, its appearance in the intracellular vesicular erythroblast compartments indicated CXCR4 endocytosis. The maximal intensity projection image at 60 min after chemokine stimulation is shown; the 3D appearance is shown in movie S3. Data are means ± SEM, and each data point represents a single mouse. Scale bars, 10 μm [(B) and (C)] and 5 μm (F).

We previously reported the binding of dimeric CXCL12 to the receptor ACKR1 (16). Consistent with this, ACKR1-positive primary mouse erythroblasts bound CXCL12. However, binding was comparable in erythroblasts from ACKR1-deficient mice (fig. S1A), suggesting the involvement of an additional high-affinity receptor. Single-cell RNA-sequencing (scRNA-seq) analysis of mouse erythroblast transcripts revealed, apart from *Ackr1*, the expression of *Cxcr4*, but not two other known CXCL12 receptors, *Ackr3* (17) and *Gpr182/Ackr5* (Fig. 1A and fig. S1, B and C) (18). *Cxcr4* transcription decreased in mature erythroblasts and was absent in reticulocytes (Fig. 1A). Furthermore, erythroblasts contained mRNA encoding Gα_i_ and β-arrestin1, downstream effectors of CXCR4, but not that encoding β-arrestin2, which was more abundant in the rest of the BM cells (fig. S1, C and D). In conventional and imaging flow cytometry, a specific anti-CXCR4 antibody (19) immunoreacted with freshly isolated primary mouse erythroblasts, confirming a gradual reduction in CXCR4 surface abundance as they matured and a lack of CXCR4 in reticulocytes (Fig. 1B and fig. S1E). In addition, the mean fluorescence intensity (MFI) values of CXCL12-AF647 binding to erythroblasts largely mirrored the extent of CXCR4 immunoreactivity at their individual maturation stages, excepting its strong binding to pyrenocytes, which are erythroblast-derived cells that contain ejected nuclei (Fig. 1C and fig. S1F). The pyrenocyte cell surface is decorated by phosphatidylserine (20), which avidly binds to CXCL12 (21). Accordingly, their binding to CXCL12 was partially inhibited by the phosphatidylserine blocker annexin V (fig. S1G). Most of the overall CXCL12 binding to erythroblasts, however, was blocked by a neutralizing anti–N-terminal CXCL12 antibody and the CXCR4 antagonists AMD3100 and Ly2510924 (Fig. 1D).

Despite engaging CXCR4 on erythroblasts, CXCL12 did not induce their migration in vitro (fig. S1H). In addition and in contrast to its effect on leukocytes, stimulation of erythroblasts with CXCL12 failed to induce rapid Ca^2+^ transients above the background of high free cytoplasmic Ca^2+^ that characterizes freshly isolated erythroblasts (fig. S1I and movies S1 and S2). However, CXCL12 rapidly reduced the abundance of CXCR4 on the erythroblast cell membrane (Fig. 1E). Confocal imaging of erythroblasts after pulse-labeling with an anti-CXCR4 antibody followed by incubation with CXCL12-AF647 revealed the internalization of CXCR4 and CXCL12 and their co-localization in multiple intracellular vesicular structures (Fig. 1F and movie S3). These mostly included Rab5-positive early endosomes, Rab7-positive late endosomes, multivesicular bodies, and, to a lesser extent, Rab11-positive recycling endosomes (fig. S2). The ligand-induced endocytosis of CXCR4 indicated that the receptor is functional in erythroblasts, prompting us to explore next its involvement in cell responses related to cell development and maturation in the erythroid lineage.

### CXCR4 signaling is required for optimal erythroblast maturation and enucleation in vivo

**Fig. 2:**
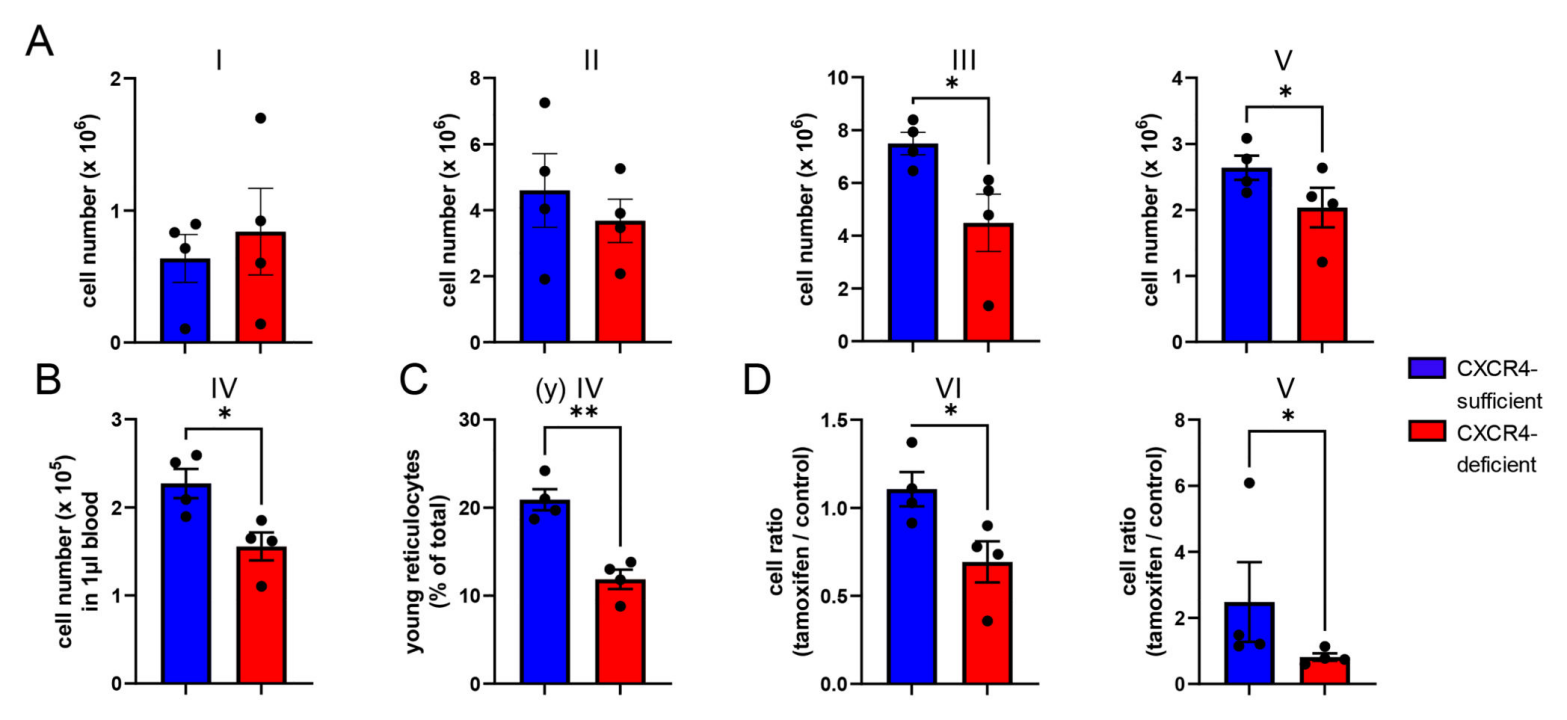
Contribution of CXCR4 to terminal erythropoiesis in vivo and in vitro. **(A)** Total numbers of murine BM proerythroblasts (I), early erythroblasts (II), late erythroblasts (III), and pyrenocytes (V), in CXCR4-deficient Cxcr4^fl/fl^ Ubc-creERT2 mice (red) and their CXCR4-sufficient control littermates (blue). (**B**) Total number of reticulocytes (IV) in the blood of CXCR4-deficient and CXCR4-sufficient control mice. (**C**) Number of young reticulocytes [(y) IV] as a percentage of total blood reticulocytes. (**D**) Number of erythrocytes and pyrenocytes in short-term cultures of erythroblasts isolated from the BM of Cxcr4^fl/fl^ Ubc-creERT2 mice and their control littermates and treated in vitro with 4-hydroxytamoxifen and CXCL12. [(A) to (D)] Cell populations were defined by flow cytometry as described for fig. S1E; young reticulocytes were identified by their high RNA content. Data are means ± SEM of four mice per genotype. *P < 0.05 by unpaired two-tailed t test.

The constitutive ablation of CXCL12 or CXCR4 is incompatible with life. Therefore, the effect of this signaling axis on BM erythropoiesis in vivo was assessed through experiments with inducible CXCR4-deficient Cxcr4^fl/fl^Ubc-creERT2 mice. We titrated the protocol of tamoxifen administration to induce a significant, albeit not complete, reduction in CXCR4 abundance selectively in erythroblasts (fig. S3, A and B). No further reduction in CXCL12 binding to erythroblasts was achieved with a CXCR4 antagonist (fig. S3C). The analysis was performed on the day after the last tamoxifen injection, before the start of stress erythropoiesis in the spleen, which developed at later times because of the loss of CXCR4 (fig. S3, D to F). Note that this protocol neither reduced the numbers of hematopoietic and stem progenitor cells (HSPCs) in the BM and spleen nor induced substantial HSPC or myeloid cell mobilization into the blood (fig. S3, G to L). Conversely, the reduction in CXCR4 abundance by tamoxifen caused changes in the numbers of BM erythroid cells. Whereas the BM counts of proerythroblasts (I) and early erythroblasts (II) remained unaffected, the numbers of late erythroblast (III) were reduced (Fig. 2A). This was not due to their mobilization from the BM (fig. S3M), potentially implicating an inhibition of their effective maturation. Furthermore, CXCR4 depletion in vivo led to a reduction in pyrenocyte (V) counts in the BM (Fig. 2A) and a reduction in the number of circulating total reticulocytes (IV) (Fig. 2B) and the proportion of their immature forms [(y) IV] (Fig. 2C), identified on the basis of their high RNA contents. This suggests that CXCR4 signaling in cells of the erythroid lineage stimulates enucleation.

To substantiate the mechanistic contribution of CXCR4 signaling to enucleation, erythroblasts and progenitor cells were isolated from Cxcr4^fl/fl^Ubc-creERT2 mice and their control Cxcr4^fl/fl^ littermates and were treated in vitro with CXCL12 and 4-hydroxytamoxifen or vehicle as a control. The Cxcr4^fl/fl^Ubc-creERT2 erythroblasts that were depleted of CXCR4 by tamoxifen produced fewer erythrocytes (VI) and pyrenocytes during their short time in culture in vitro as compared to the identically treated erythroblasts of control mice (Fig. 2D). In addition to using CXCR4-depleted Cxcr4^fl/fl^Ubc-creERT2 mice, we used wild-type (WT) mice to evaluate the effect of a short-term treatment in vivo with a CXCR4 antagonist on erythroblast populations in the BM. Three hours after the bolus injection of Ly2510924, we observed an accumulation of proerythroblasts and a reduction in the numbers of late erythroblasts (fig. S3N). These findings are consistent with the CXCR4 blockade interfering with terminal erythropoiesis.

### Differentially expressed genes in CXCR4-sufficient and CXCR4-deficient erythroid cells are involved in metabolism and chromatin organization

**Fig. 3:**
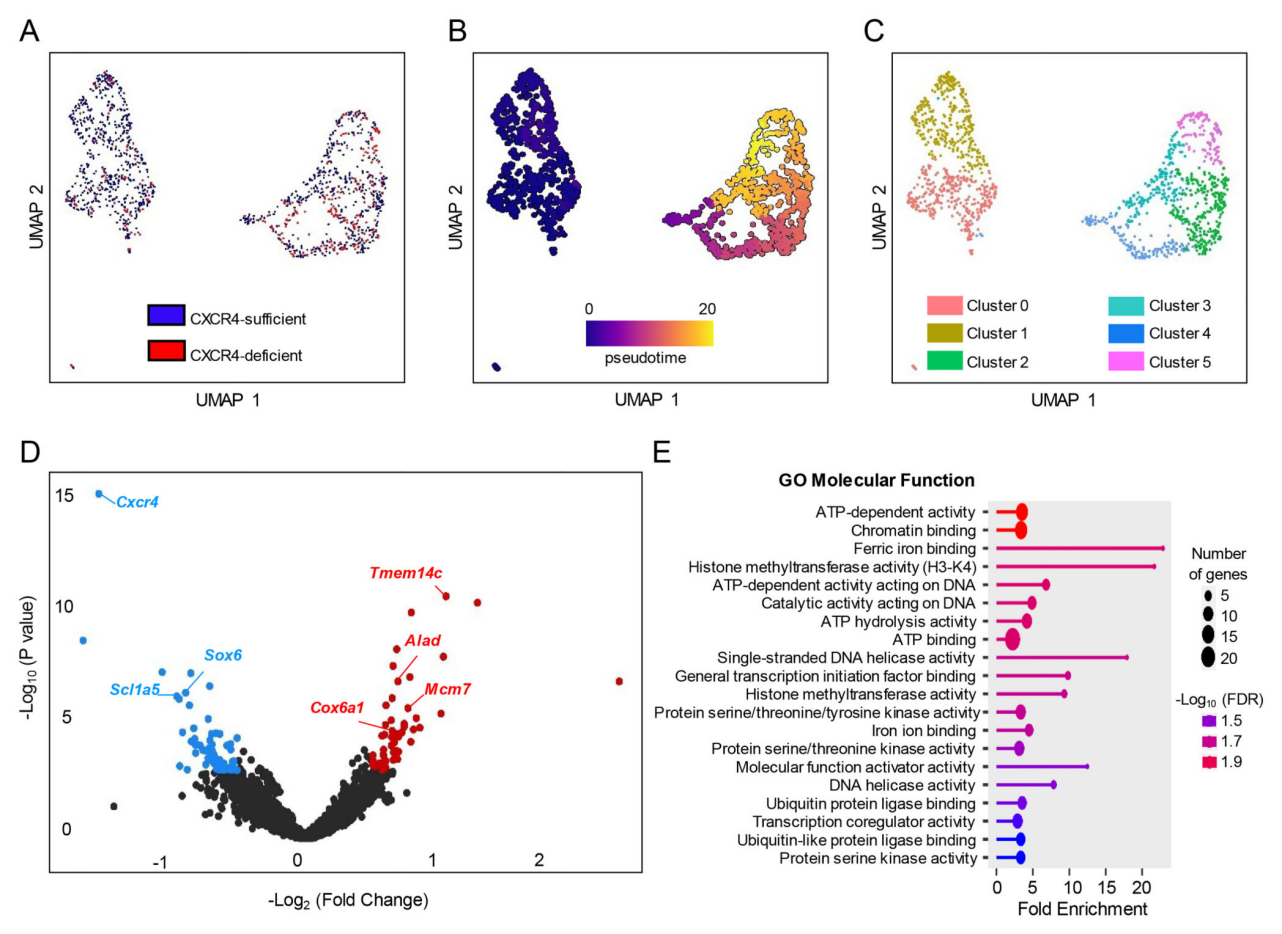
Differentially expressed genes in CXCR4-sufficient versus CXCR4-deficient erythroblasts are involved in metabolism and nucleus organization. **(A)** UMAPs of combined data from the scRNA-seq analysis of CXCR4-sufficient (blue) and CXCR4-deficient (red) mouse CD71^+^ BM cells. (**B**) Pseudotime trajectory analysis of combined data from the scRNA-seq analysis of CXCR4-sufficient and CXCR4-deficient CD71^+^ BM cells. (**C**) Six clusters were identified, grouped as progenitors (clusters 0 and 1) and erythroblasts (clusters 2 to 5), according to pseudotime analysis and gene expression as seen in fig. S4A showing *Cd34, Cd44*, and *Tfrc* expression in progenitor clusters and *Tfrc, Gata1, Gypa*, and *Hbb-bt* in erythroblast clusters. (**D**) Volcano plot of differentially expressed genes in erythroblasts (clusters 2 to 5). Genes that were decreased in expression in CXCR4-deficient cells with a log_2_ fold change (log_2_FC value of <−0.5 are in blue, whereas genes that were increased in expression in CXCR4-deficient cells with a log_2_FC value > 0.5 are in red. (**E**) GO molecular function analysis of all of the differentially expressed genes that showed a log_2_FC value of >0.5 and <−0.5 in CXCR4-deficient CD71^+^ BM cells (clusters 2 to 5). ATP, adenosine 5′-triphosphate; FDR, false discovery rate

To investigate whether physiological CXCR4 signaling in erythroblasts affected their gene expression, we compared the individual transcriptomes of the cells broadly belonging to the erythroid lineage in Cxcr4^fl/fl^Ubc-creERT2 and control Cxcr4^fl/fl^ mice. Animals were treated with tamoxifen as described earlier, CD71^+^ BM cells were isolated, and their complete transcriptomes were captured and analyzed by scRNA-seq. The resultant datasets were combined and clustered in a uniform manifold approximation and projection (UMAP), which delineated two main cell groups (Fig. 3A). Pseudotime analysis identified the differentiation course within the erythroid populations (Fig. 3B), predicting a trajectory from erythroid progenitors (clusters 0 to 1) to erythroblasts (clusters 2 to 5) (Fig. 3C and fig. S4, A and B).

The analysis of differentially expressed genes in CXCR4-sufficient versus CXCR4-deficient erythroblast populations (clusters 2 to 5) showed that *Cxcr4* was the gene most significantly reduced in expression (Fig. 3D and data file S1). Conversely, tamoxifen failed to significantly reduce the number of *Cxcr4* transcripts in the progenitor populations, which may explain why their numbers remained unaffected when using the short-term protocol and underscored why it was ideally suited to evaluate CXCR4 involvement selectively in terminal erythropoiesis. In addition to *Cxcr4*, 92 genes were decreased in expression in CXCR4-depleted erythroblasts with a value for the log of the fold change in expression (logFC) < 0.50 and a false discovery rate < 0.05 (*P* < 0.001).

Overall, the proteins encoded by the differentially expressed genes are involved in chromatin organization and transcriptional regulation, as well as metabolic processes (Fig. 3E). Panther gene ontology (GO) cellular component enrichment analysis showed that 59 of the proteins encoded by the genes reduced in expression in CXCR4-depleted erythroblasts function in the nucleus, whereas enriched GO biological processes contained chromatin organization (data file S1 and fig. S4, C and D). *Sox6*, which encodes Sox6, the transcription factor involved in the regulation of globin gene expression in definitive erythroid cells (22), was one of the genes most significantly reduced in expression in CXCR4-deficient erythroblasts (Fig. 3D and data file S1). Several genes that encode proteins involved in cellular transport were also reduced in expression, including *Scl1a5* (Fig. 3D and data file S1). It encodes SLC1A5 (ASCT2 glutamine transporter), which contributes to erythroid lineage specification and erythroid differentiation (23). Using the same statistical thresholds, we delineated 58 genes that were increased in expression in CXCR4-deficient erythroblasts (data file S1), with 44 and 15 proteins encoded by these genes localizing in the cytoplasm and mitochondria, respectively (data file S1). The most enriched GO molecular function was ferric iron binding. GO biological process analysis revealed a high enrichment of genes involved in nuclear DNA replication; heme biosynthesis process, for example, *Tmem14c* and *Alad*; as well as aerobic respiration, for example, *Cox6a1* (Fig. 3D and data file S1). This signature is consistent with CXCR4-depleted erythroblasts switching on compensatory transcriptional programs in heme production and proliferation. Together, these scRNA-seq findings suggest that CXCR4 in erythroblasts promotes key transcriptional programs in terminal erythropoiesis, particularly those related to intranuclear processes.

### Endocytosed CXCR4 is transported onto the nuclear envelope and into the nucleus and activates a specific subset of serine/threonine kinases

**Fig. 4:**
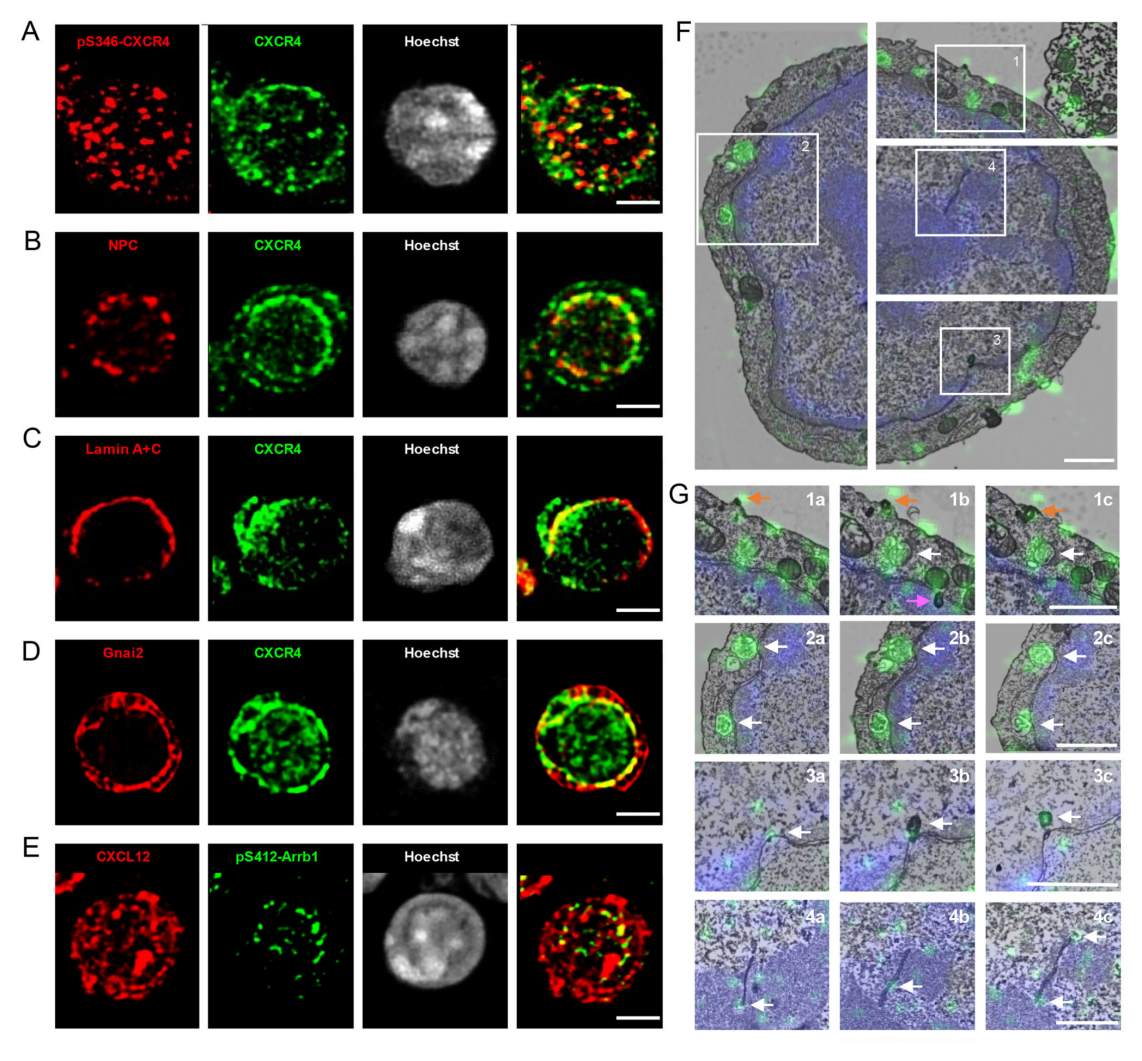
Intracellular localization of CXCL12 and CXCR4 in erythroblasts. (**A** to **E**) Confocal immunofluorescence microscopy images of 0.2- to 0.5-μm optical sections. Freshly isolated murine BM erythroblasts stimulated with CXCL12 and stained with anti-CXCR4 antibody (green) and co-stained with (A) anti–pS346 CXCR4 antibody (red); see the 3D appearance in movie S4; (B) anti–nuclear pore complex (NPC) antibody (red); (C) anti–lamin A + C antibody (red); or (D) anti-Gαi_2_ antibody (red). (E) Freshly isolated murine BM erythroblasts stimulated with CXCL12-AF647 (red) and co-stained with anti–pS412 Arrb1 antibody (green). (**F**) Correlative immunofluorescence–electron microscopy of a freshly isolated erythroblast stimulated with CXCL12, pulsed with anti–CXCR4-AF488 (green), and counterstained with Hoechst (blue). The image was obtained by aligning 0.3-μm optical confocal sections of the cell with images of its 0.07-μm serial sections taken after processing for ultrastructural observation under the electron microscope. Four segments show different *z*-planes of the same erythroblast, representative of eight that were investigated. Separated fluorescent and electron microscopy images are shown in fig. S7 (A to D). (**G**) Three sequential ultrastructural images aligned with their corresponding confocal optical sections defined in four marked areas in (F). Area 1: CXCR4 endocytosis from the cell membrane (1a to 1c; orange arrows); CXCR4-containing large multivesicular bodies in close proximity to the nuclear membrane (1b and 1c; white arrows); and CXCR4-containing membrane invagination from the nuclear envelope into the nucleus (1b; pink arrow). Area 2: CXCR4-containing multivesicular bodies fused with the outer layer of nuclear envelope (2a to 2c; white arrows). Area 3: Invagination of a CXCR4 positive segment of the nuclear envelope into the nucleus (3a to 3c; white arrows). Area 4: CXCR4-associated fluorescent signal within the nucleoplasmic reticulum (4a to 4c; white arrows) and not associated with any discernable ultrastructure in the nucleoplasm. Scale bars, 5 μm [(A) to (E)] and 1 μm [(F) and (G)].

After stimulation of erythroblasts with CXCL12, the endocytosed CXCR4 was phosphorylated at Ser^346^ and, in ~30% of cells, appeared unexpectedly apart from the cytoplasm, also prominently in the nucleus (Fig. 4A and movie S4). The anti–pS346/347 CXCR4 antibody that we used was previously validated in experiments with mock-transfected human embryonic kidney 293 cells and transfected cells expressing WT S346-348A mutant CXCR4 (24). CXCR4 phosphorylation was reduced by the CXCR4 antagonist and PTX (fig. S5, A and B). Unstimulated erythroblasts also contained basal amounts of phosphorylated CXCR4, including in the nucleus, likely in response to endogenous CXCL12 bound to erythroblasts before their isolation from the CXCL12-rich BM (fig. S5, B to D). In addition to appearing in erythroblast nuclei, CXCR4 was observed in some cells particularly enriched on the nuclear membrane. Here, it co-localized with proteins of the nucleopore complex and lamins A and C (Fig. 4, B and C) and with Gαi_2_ (Fig. 4D), which, however, remained entirely excluded from the nucleus. Conversely, phosphorylated β-arrestin1 was also prominently localized in the nucleus itself (Fig. 4E). Together, the perinuclear and nuclear co-localization of CXCL12, CXCR4, and its two main molecular effectors, the G protein Gαi_2_ and β-arrestin1, suggests ongoing intracellular CXCR4 signaling within these compartments.

Next, we explored whether CXCR4 stimulated signaling pathways in erythroblasts involve specific protein kinases, a ubiquitously expressed group of enzymes that phosphorylate intracellular effector proteins and promote multiple cell functions. To this end, we performed a comprehensive screen of serine/threonine kinase activities in erythroblasts that were freshly isolated from the BM of CXCR4-deficient Cxcr4^fl/fl^Ubc-creERT2 mice or their CXCR4-sufficient littermates. The activities of multiple kinases, primarily of AGC family [including cyclic adenosine 3′,5′-monophosphate–dependent protein kinase (PKA), cyclic guanosine 3′,5′-monophosphate–dependent kinase (PKG), and protein kinase C (PKC)] and inhibitory κB kinase α (IKKα), were substantially depleted in CXCR4-deficient erythroblasts (fig. S5E and data file S1). Some of these enzymes, for example, PKA, have been implicated in erythroblast responses during terminal erythropoiesis (25), but most have not been previously linked with the erythroid lineage. Several of the activated kinases localize to and act in the nucleus, albeit mostly in cancer cells, including proto-oncogene serine/threonine-protein kinase (Pim-1) (26, 27), protein kinase X (PrKX) (28), NIMA related kinase 10 (Nek10) (29).

We then compared kinase activities in CXCR4-sufficient erythroblasts at 10 min after ex vivo stimulation with CXCL12 with that after 60 min, when CXCR4 was mostly localized intracellularly in vesicular perinuclear and nuclear compartments. At 60 min after stimulation, several AGC- kinases and IKKα were substantially more active than at 10 min, and their fingerprint very closely mirrored that activated in CXCR4-sufficient as compared to that in CXCR4-deficient erythroblasts (fig. S5F and data file S1). These findings are consistent with the notion that CXCR4 signaling from intracellular compartments activates AGC kinases and IKKα. This characteristic set of kinases was not changed in CXCR4-deficient erythroblasts at 60 min as compared with 10 min of CXCL12 stimulation, whereas several other kinases were at a lower activation level (fig. S5G and data file S1). Together, these data indicate that multiple PKA-, PKG-, and PKC-dependent signaling pathways are stimulated in erythroblasts downstream of CXCR4 ligation by CXCL12 when both the receptor and the chemokine localize in the intracellular vesicular cell structures and the nucleus, thus again suggesting ongoing CXCR4 signaling from these compartments.

A combination of two electron microscopy approaches identified intracellular erythroblast organelles harboring CXCR4 and outlined the steps of its translocation from the cell membrane to the nucleus. Freshly isolated primary erythroblasts were stimulated with CXCL12 and pulse-labeled with fluorescent anti-CXCR4 antibody. The subcellular localization of the latter was detected by correlative fluorescence and electron microscopy (Fig. 4, F and G) and complementarily by immuno–electron microscopy with a peroxidase-labeled secondary antibody and diaminobenzidine (DAB) as a substrate, resulting in deposition of an electron-dense precipitate (figs. S6 and S7). Apart from individual endosomal vesicles budding from the erythroblast membrane (Fig. 4G, region 1, and fig. S6A, region 1), CXCR4 prominently appeared within the large multivesicular bodies (Fig. 4G, regions 1 and 2; fig. S6A, regions 2 and 3; and fig. S6B), an organelle that increases in number during late erythroblast maturation (30). Some of the multivesicular bodies were observed in direct contact and fusing with the outer layer of the nuclear envelope (Fig. 4G, region 1, and fig. S6C), consistent with vesicular transport of CXCR4 as a apotential mechanism of its translocation onto the nuclear envelope and into the nucleus, which is proposed as the main transport route for other cell membrane proteins (31) but, hitherto, has not been shown for CXCR4 or any other chemokine receptor, neither in erythroblasts nor in other cells. The CXCR4 signal within the nucleus itself was visible in discrete invaginations of the nuclear membrane (Fig. 4G, regions 1 and 3) and appeared associated with the components of the nucleoplasmic reticulum (Fig. 4G, region 4, and fig. S6D), a nuclear organelle formed by deep folds of the nuclear envelope (32, 33), which, hitherto, has not been noted in erythroblasts. In some cases, the CXCR4-associated signal could not be attributed to a recognizable nuclear ultrastructure (Fig. 4G, region 4). Less frequently, CXCR4 was associated with membranous structures in the cytoplasm and mitochondria (fig. S7, E and F).

### CXCL12 stimulates the polarization and enucleation of erythroblasts

**Fig. 5:**
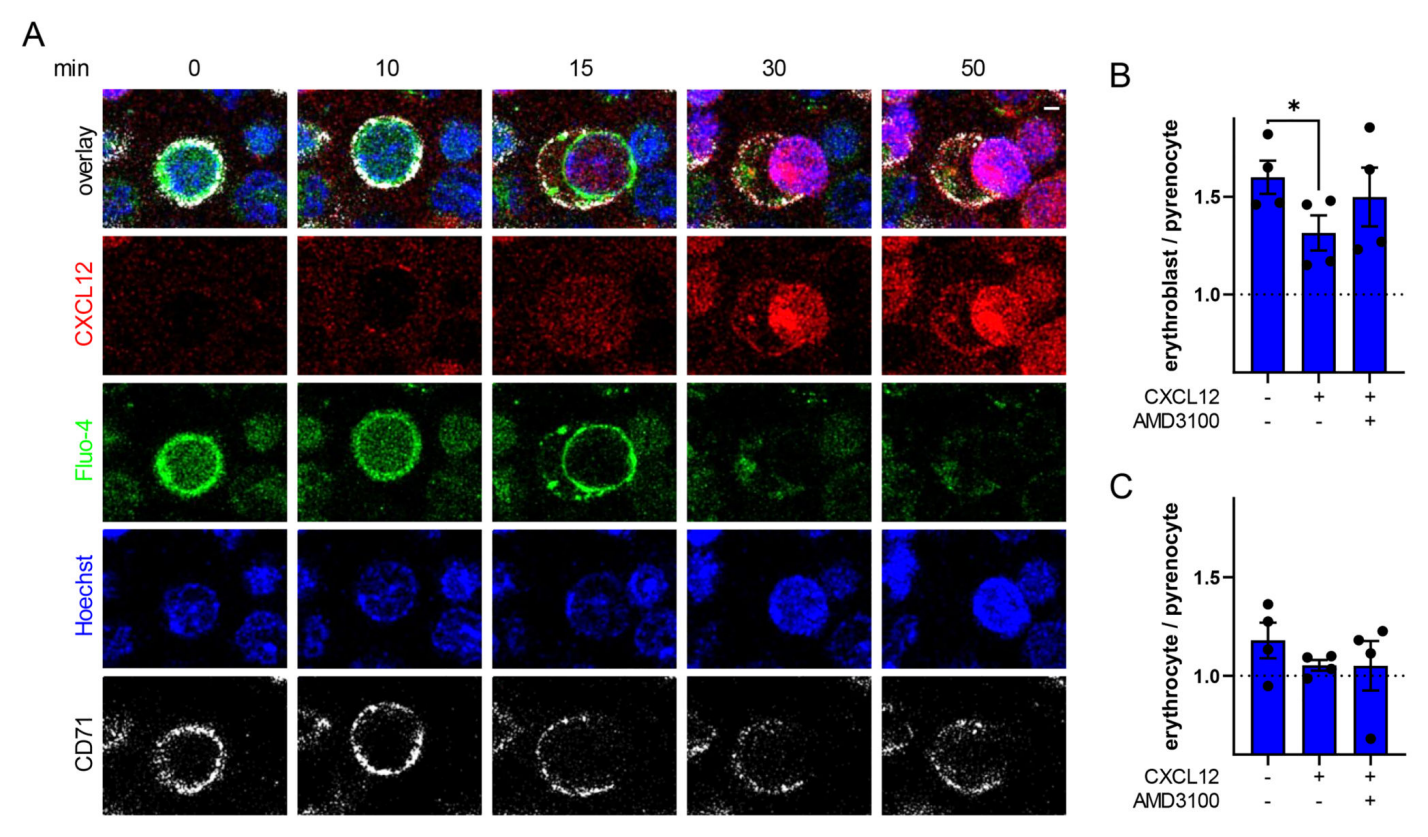
CXCL12-stimulated erythroblast elongation, nuclear polarization, chromatin condensation, and enucleation. **(A)** Murine BM-derived erythroblasts were pre-labeled with the Ca^2+^ sensitive dye Fluo-4 (green), anti-CD71 antibody (white), and the nuclear dye Hoechst (blue), which was followed by CXCL12-AF647 (red) that was added during live-cell imaging recorded under a confocal microscope. Images shown were taken at 0, 10, 15, 30, and 50 min. Scale bar, 2 μm. The time-lapse sequence is shown in movie S6. (**B** and **C**) Freshly isolated erythroblasts were treated with CXCL12 alone or with the CXCR4 antagonist AMD3100. Cell ratios of erythroblasts and pyrenocytes (B) and enucleated erythrocytes and pyrenocytes (C) as enumerated by imaging flow cytometry at the end of overnight in vitro erythroblast cultures. Data are means ± SEM. Data points represent separate experiments on erythroblasts from individual mice (*n* = four mice). *P < 0.05 by one-way ANOVA with Tukey’s multiple comparisons test.

To substantiate any involvement of CXCR4 in erythroblast enucleation, which was suggested in vivo and in vitro (Fig. 2, B to D), we investigated the subcellular dynamics of erythroblast responses to CXCL12. Time-lapse microscopy showed that, in response to CXCL12, a subset of freshly isolated erythroblasts became elongated and their nuclei shifted to one side, reduced in size, and compacted their chromatin, which did not occur in the absence of CXCL12 (fig. S8, A and B, and movie S5). Imaging flow cytometry recorded the cell morphological parameters for the entire broadly diverse XCL12 principally to T of erythroblasts at different maturation stages and ascribed numerical values to the CXCL12-induced changes. Upon stimulation, ~30% more erythroblasts became ellipsoid and dislocated and condensed their nuclei (fig. S8, C to E). All CXCL12-induced changes in erythroblasts were blocked by a CXCR4 antagonist (fig. S8, C to E). During the CXCL12-induced polarization of erythroblasts, the distribution pattern of cytosolic free Ca^2+^ changed from being diffuse and cytoplasmic to a short wave of intense perinuclear pattern, potentially reflecting CXCR4 signaling from this subcellular compartment, followed by the disappearance of free Ca^2+^ from erythroblasts (Fig. 5A and movie S6). After the perinuclear Ca^2+^ wave, the erythroblast nuclei moved to an eccentrical position in the cell and simultaneously accumulated CXCL12 (Fig. 5A and movie S6), whereas the cell halves that harbored nuclei became enriched in CXCR4 (fig. S8A and movie S5).

The observed CXCL12-induced morphological changes in erythroblasts, including their elongation and the asymmetry and dislocation of their nuclei, immediately precede erythroblast enucleation and its division into a nucleus-containing pyrenocyte and an anuclear reticulocyte (9). Also during a short-term assay in vitro, the addition of CXCL12 to freshly isolated erythroblasts increased enucleation, as manifested in increased number of pyrenocytes (Fig. 5, B and C), when compared to unstimulated erythroblasts. Substantial baseline enucleation levels observed in the latter potentially reflected their responses to background amounts of CXCL12 that were inherently carried by erythroblasts ex vivo from the BM (fig. S5, C and D), where CXCL12 is abundantly constitutively produced (7). Dissimilar in vivo exposure of primary erythroblasts to microenvironment-dependent amounts of CXCL12 in the BM, in addition to their dynamic maturational heterogeneity, contributes to a variation in erythroblast in vitro responses to CXCL12. The recorded different morphological patterns of intracellular CXCL12/CXCR4 localization in erythroblasts reflected their broadly heterogeneous nature in the BM as they asynchronously matured and represented different stages of erythroblast development. Nuclear localization of CXCL12 appeared more prominent in the large loose nuclei of immature erythroblasts (fig. S8F). Accordingly, imaging flow cytometry ascribed the intranuclear localization of CXCL12 principally to Ter119^low^CD71^high^ early erythroblasts, whereas the cytoplasmic extranuclear and nuclear membrane localization marked more Ter119^high^CD71^low^ late erythroblasts (fig. S8, G to I).

Together, these findings suggest that the CXCL12-induced, CXCR4-mediated signals in erythroblasts promote an alternative set of cell-characteristic functional responses, which, instead of mediating conventional cell migration, entail morphological changes culminating in erythroblast division into anuclear reticulocytes and nucleus-containing pyrenocytes.

## Discussion

We observed the expression of CXCR4 in erythroblasts and discovered its previously uncharacterized association with their nuclear envelope and nucleus, where it stimulated distinct cell-type–specific responses promoting erythroblast maturation and enucleation. In addition, CXCL12 acted as a cell-exogenous mediator through its cognate G protein–coupled receptor (GPCR) to stimulate Ca^2+^ transients, activate a subset of kinases, and induce erythroblast changes, leading to their enucleation. These activities potentially place CXCL12 signaling upstream of intracellular effectors, such as diaphanous-related formin mDia2 and small guanosine triphosphatases, which promote erythroblast enucleation (34) but, in other cell types, mediate CXCR4-dependent cytoskeletal changes during migration (35). This sets CXCL12 apart from other molecules required for erythroblast maturation, for example, EPO, GATA1, and KLF1 (36, 37). Unlike CXCR4, a GPCR that stimulates immediate cytoskeletal changes in response to CXCL12, multiple cytokines and growth factors signal through either type 1 cytokine receptors or tyrosine kinase receptors, eliciting much slower responses that take several hours to days to develop. These mainly involve erythroblast proliferation, transcription, metabolism, and anti-apoptotic signaling. Naturally, stimuli that induce erythroblast differentiation subsequently also promote the development of chromatin condensation, nuclear dislocation, and, ultimately, enucleation, although they cannot do so directly (10, 38).

In clinical samples of various cancers, CXCR4 immunoreactivity has also been detected in cell nuclei. Largely lacking specificity controls, such nuclear CXCR4 immunoreactivity potentially reflects immunostaining artifacts (39) that commonly plague anti–chemokine receptor antibodies (40). In some cases, the frequency of nuclear CXCR4 immunoreactivity in cancer cells correlated with a more malignant tumor behavior, albeit due to entirely unexplored mechanisms (41–46). It is possible that erythroid-specific cell programs that drive the nuclear localization and signaling of CXCR4 could be spuriously activated in malignant cells. However, because CXCR4 is normally expressed in cells of many ontologies, it is equally possible that intranuclear CXCR4 localization and signaling also occur in cells other than erythroblasts, inducing their atypical, hitherto, uncharacterized cell responses that potentially are recapitulated in the malignancies emerging from these cells.

Note that intracellular and intranuclear signaling has been observed for some non-chemokine GPCRs, with potential differential cell responses mediated from these compartments (47, 48). For example, the prototypical GPCR, the β2-adrenoceptor, is active and signals through G proteins not only from the plasma membrane but also from endosomes (49). Similarly, chemokine ligation of some chemokine receptors, for example, CCR7, leads to the formation of endomembrane-residing signaling complexes (50). One report in cancer cells described CXCR4 endocytosis as a requirement for optimal CXCR4-promoted AKT activation and signaling (51). We found that a specific set of kinases was activated after CXCL12/CXCR4 internalization in erythroblasts; however, the detailed signaling mechanisms downstream of G protein activation and β-arrestin engagement remain to be elucidated. Furthermore, internalized CXCL12 co-localized in the nucleus with phosphorylated β-arrestin1, the predominant isoform found in erythroblasts. β-Arrestin1, but not β-arrestin2, was described to locate in the nucleus with a potential role in chromatin organization (52). Note that these two β-arrestin isoforms can mediate distinct cell responses downstream of CXCR4 (53, 54), potentially explaining why the observed intracellular trafficking of CXCR4 in erythroblasts and their ensuing responses to CXCL12 were markedly different from the well-established responses in leukocytes.

**Fig. 6:**
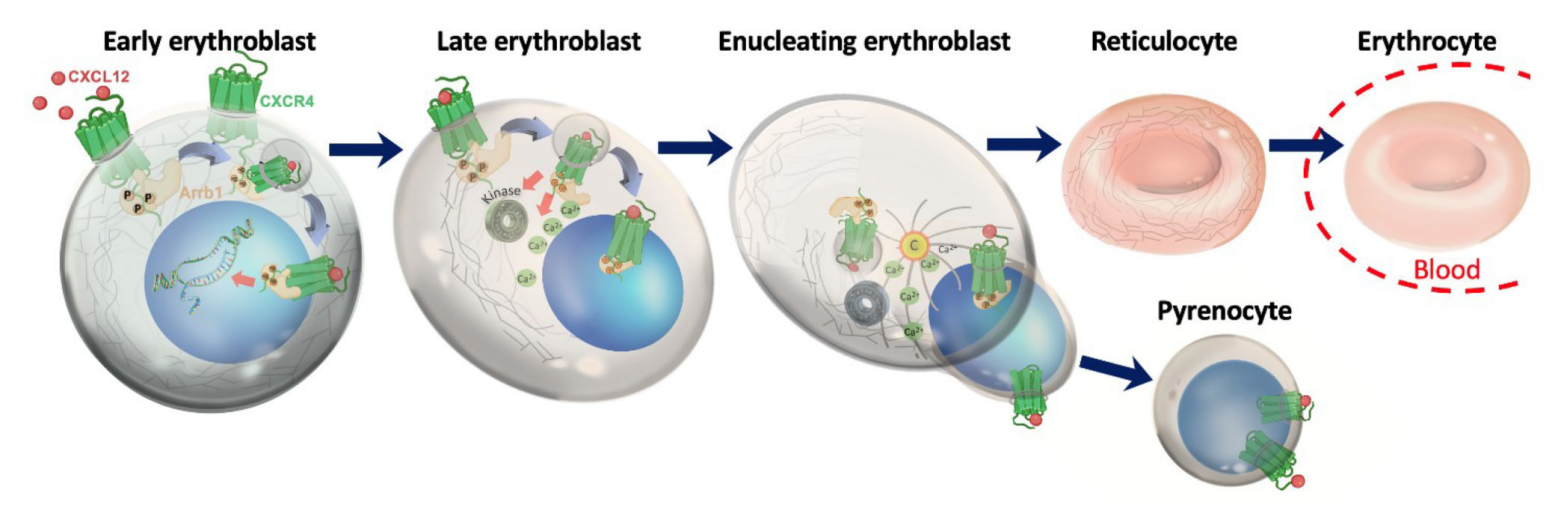
The role of CXCR4 signaling in erythropoiesis. Erythroblasts express CXCR4 and bind to CXCL12 but fail to migrate toward it. Instead CXCL12, CXCR4, and β-arrestin1 are co-internalized and localize around or in the nucleus where they remain for a prolonged time. CXCL12 signaling in erythroblasts (i) changes transcription, (ii) accelerates late erythroblast maturation, (iii) induces cell and nuclear polarization, (iv) activates kinases, (v) causes Ca^2+^fluxes in the perinuclear areas, and (vi) promotes enucleation. Upon erythroblast division into a reticulocyte and a pyrenocyte, CXCR4 is sorted entirely into the pyrenocyte.

Morphological features of the sequential subcellular processes that precede and accompany erythroblast enucleation have been described in detail (9). Conversely, the molecular cues that stimulate these processes have remained elusive. We showed that CXCL12, a chemokine constitutively produced in the BM, signaled through CXCR4 to promote erythroblast enucleation by inducing erythroblast asymmetry, polarization of functional surface molecules, nuclear condensation, and its extreme excentric dislocation, leading to enucleation (Fig. 6). However, additional stimuli might be required to optimally achieve complete separation of pyrenocytes from reticulocytes. In vivo, such stimuli might come from the subset of macrophages that organize erythroblastic islands (55), support enucleation, and rapidly phagocytose pyrenocytes (56). The latter process might be facilitated by CXCL12 binding to erythroblasts as mediated by either CXCR4 or phosphatidylserine (57).

Our discovery of the critical role the CXCL12-CXCR4 axis plays in erythropoiesis should instruct future studies investigating its potential dysregulation in pathologies affecting erythropoiesis and may lead to the development of new targeted therapeutic approaches in these diseases. Moreover, our data suggest the use of CXCL12 to improve the efficacy of the in vitro production of erythrocytes from stem cells and engineered precursor cells. Such highly promising technological approaches aimed at circumventing the use of donated blood (58) are now hampered by ineffectual terminal erythropoiesis in vitro, insufficient erythroblast enucleation in particular (59), thus preventing their widespread practical utility.

## Materials and Methods

### Mice

WT C57BL/6J and UBC-Cre-ERT2 mice were purchased from Charles River Laboratories. The Cxcr4-floxed (Cxcr4^fl/fl^) mice were provided by Y. Zou (Columbia University, New York, USA). ACKR1-deficient mice were generated as described previously (60). All mice were group housed in individually ventilated cages under specific pathogen–free conditions with a 12-hour light-dark cycle and were used at 2 to 4 months of age. Animals were humanely culled by CO_2_ and cervical dislocation in accordance with UK Home Office regulations. All in vivo experiments were conducted at the William Harvey Research Institute, Queen Mary University of London, UK, under UK legislation for animal experimentation and in agreement with the UK Home Office Animals Scientific Procedures Act 1986.

### Cre induction for in vivo CXCR4 depletion

The tamoxifen-inducible conditional Cxcr4^fl/fl^ UBC-Cre-ERT2 mice were used as a CXCR4-deficient strain, and their littermates, Cxcr4^fl/fl^ or CXCR4 WT UBC-Cre-ERT2, were used as CXCR4-sufficient controls. For a stock solution of tamoxifen, its free base (Sigma-Aldrich, T5648) was dissolved in 100% (v/v) ethanol and diluted in peanut oil (Sigma-Aldrich, P2144) to a final concentration of tamoxifen (10 mg/ml), 25% ethanol, and 75% peanut oil. The stock was stored at 4°C for up to 3 days in the dark. Before injection, the tamoxifen stock was diluted 1:1 with peanut oil and vortexed vigorously to give a final working concentration of 5 mg/ml. Tamoxifen solution (100 μl) was injected intraperitoneally per mouse on 3 consecutive days, and tissues were harvested and analyzed on the day after the last injection (fig. S5A).

### 3′ RNA library preparation and scRNA-seq

BM cells were depleted of CD11b^+^, CD5^+^, B220^+^, and Ly6G^+^ cells by magnetic cell sorting (Miltenyi Biotec) and stained with anti-CD71, anti-Ter119, as well as for lineage markers with anti-CD11b, anti-CD19, anti-CD3, and anti-Gr1 antibodies. All antibodies and their provenances are listed in table S1. The Lin^−^CD71^+^Fsc^int/hi^ cell population was fluorescence-activated cell sorting sorted on a FACSAria II flow cytometer (BD Biosciences), fixed in 80% methanol on ice, and stored at −80°C. Single-cell RNA libraries were generated with the Chromium Single Cell 3′ kit (10x Genomics). The cells were counted by trypan blue exclusion count assay with a hemocytometer and diluted for loading onto the Chromium Controller. Loading was performed to target capture of ~3000 GEMs per sample for downstream analysis, and samples were processed through the Chromium Controller according to the standard manufacturer’s specifications. The sequencing libraries were evaluated for quality on the Agilent TapeStation (Agilent Technologies) and quantified with a Qubit 2.0 Fluorometer (Invitrogen). Pooled libraries were quantified by quantitative polymerase chain reaction (qPCR; Applied Biosystems) before being loaded onto an Illumina sequencing platform. The samples were sequenced at a configuration compatible with the recommended guidelines as outlined by 10x Genomics. Raw sequence data (.bcl files) were converted into fastq files and de-multiplexed using the 10x Genomics’ cellranger mkfastq command. Subsequent unique molecular identifier (UMI) and cell barcode deconvolution, together with mapping to respective genomes, were performed using 10x Genomics’ Cell Ranger software package to generate the final digital gene expression matrices and cloupe files. UMAP graphs were generated with the Loupe Browser 6 software (10x Genomics), and the B cell cluster defined by *Ebf1* and *Cd79a* expression was excluded.

### scRNA-seq data analysis

Cell Ranger (v3.1.0 pipeline version) was used to analyze the data generated by Single Cell 3′ RNA-seq (10x Genomics; https://support.10xgenomics.com/single-cell-gene-expression/software/overview/welcome). Briefly, the pipeline demultiplexes raw base call files generated by the sequencing in FASTQ files and then aligns [performed with STAR software (61) and the *Mus musculus* genome (GRCm38) as the reference genome], filters, and counts (barcode-processing and UMIs) the reads. This pipeline generates a matrix with only detected cell-associated barcodes with rows corresponding to features (genes) and barcode sequences (one for each cell) as columns. Each element of the matrix is the number of UMIs associated with a feature and a barcode. Before data analysis, the Seurat R package (62) was used to filter low-quality cells, including cells with unique feature counts of >4000 (cell doublets or multiples) or <250 (empty droplets). CD71+ BM-derived cells from CXCR4-sufficient and CXCR4-deficient mice were aggregated, and B cell contamination was removed (*Cd79a* and *Cd79b*), which was followed by feature expression normalization, principal components analysis, dimensionality reduction, and clustering analysis performed with the Seurat R package. Data visualization was performed with the UMAP algorithm and the principal components that explained at least 2% of the variance. Differential gene expression analysis (between CXCR4-sufficient and CXCR4-deficient cells) was performed after cell identification by *K*-means clustering and choosing the erythroblast cluster with high expression of *Gypa* and *Hbb-bt* and low expression of *Cd34* and *Cd44*. The likelihood ratio test from Edge R (Bioconductor R package; https://bioconductor.org/packages/devel/bioc/html/edgeR.html). Good gene expression was considered if at least two cells contained more than two transcripts from the gene. Statistical significance was considered with an adjusted (Benjamini-Hochberg procedure correction) *P* value of <0.05. Cell trajectory analysis was performed with the Monocle 3 R package (https://cole-trapnell-lab.github.io/monocle3/) using the UMAP method reduction and identifying the progenitor clusters (cluster 0; high expression of Cd34) as the root cells. Enrichment analysis was performed with ShinyGO 0.8 using GO data (https://zenodo.org/records/10536401) (63). The scRNA-seq data are deposited at GEO with accession number GSE272318.

### Quantitative polymerase chain reaction

RNA isolation was performed with an RNeasy Mini Kit (QIAGEN), and cDNA synthesis was performed with SuperScript VILO (Invitrogen, 11754050). qPCR was performed on the Applied Biosystems QuantStudio 7 Flex Real-Time PCR system (Applied Biosystems) using TaqMan Gene Expression Assays. Results were quantified by normalization to *Gapdh* expression using the ΔCT method.

### Flow cytometry

To obtain BM cells, femora, tibiae, and humeri were flushed with phosphate-buffered saline (PBS), and cells were filtered through a 70-μm strainer. Total BM cells were quantified in all six bones used. To obtain splenocytes, whole spleens were crushed through a 70-μm cell strainer in PBS. Blood was taken by cardiac puncture and mixed 1:10 with 0.1 M EDTA. For blood counts of erythroid lineage, cells were analyzed in 1 μl of whole blood. For blood counts of myeloid cells and stem and progenitor cells, 100 μl of whole blood were lysed with Red Blood Cell Lysis Buffer (Abcam, ab204733) for 15 min at room temperature, vortexed, and washed with PBS. For the enumeration of the erythroid lineage, the cells were stained with antibodies against CD71, Ter119, and CD45 and with Hoechst 33342 (Abcam, ab228551). Erythroid cells at different stages of erythropoiesis were defined as follows: CD45^−^ and CD71^hi^Ter119^int^Hoechst^int^ (I, proerythroblasts), CD71^hi^Ter119^hi^Hoechst^int^Fsc^hi^ (II, early erythroblasts), CD71^hi^Ter119^hi^Hoechst^int^Fscint (III, late erythroblasts), CD71^int^Ter119^hi^Hoechst^neg^Fsc^10^ (IV, reticulocytes), CD71^int^Ter119^lo^Hoechs^thi^ (V, pyrenocytes), and CD71^lo^Ter119^hi^Hoechst^neg^Fsc^lo^ (VI, mature erythrocytes). Details of the gating strategy used are shown in fig. S1E. In addition, total BM leukocytes were defined as CD45^+^CD71^−^Ter119^−^ cells, Lin^−^Sca^−^1+c-kit^+^ (LSK cells), Lin^−^c-kit^+^ myeloid progenitors (MP cells), CD45^+^CD11b^+^Ly6G^+^CD115^−^ (neutrophils), and CD45^+^CD11b^+^CD115^+^Ly6G^−^ (monocytes), as previously described (15). CXCR4 expression was assessed as the cell surface immunoreactivity in the cell populations and expressed as specific MFI. In all flow cytometry experiments, antibody staining was performed at 4°C for 20 min, and the cells were washed and kept on ice in PBS until freshly analyzed. Cells were counted with CountBright Absolute Counting Beads (Life Technologies) and analyzed with either an LSRFortessa flow cytometer (BD Biosciences) or a Symphony A3 flow cytometer (BD Biosciences), and data were analyzed with Kaluza software (Beckman Coulter).

### Blood reticulocyte counts

Mouse blood was taken by cardiac puncture and mixed 1:10 with 0.1 M EDTA. Reticulocyte nucleic acid content defining reticulocyte age and other blood parameters were determined with an automated Procyte Dx blood counter (IDEXX).

### Alexa Fluor 647–conjugated CXCL12 binding and CXCR4 internalization

BM cells were incubated with 100 nM CXCL12-AF647 (Almac, CAF-50) containing PBS for 45 min at room temperature with or without a 10-min pretreatment with 10 μM AMD3100 (Abcam, ab120718), 10 μM Ly2510924 (Tocris, 3097), or anti–CXCL12 N-terminal antibody (30 μg/ml; Sigma-Aldrich, clone K15C). annexin V binding competition was performed in annexin V binding buffer (BioLegend) or PBS, with cells pretreated with annexin V–fluorescein isothiocyanate (FITC) for 5 min at room temperature before CXCL12-AF647 was added. For CXCR4 internalization experiments, BM cells were incubated with 100 nM CXCL12 (Peprotech, 250-20A) in Iscove’s modified Dulbecco’s medium (IMDM; Gibco, 12440053) for 45 min at 37°C with or without a 10-min pretreatment with 10 μM AMD3100. The cells were then washed with cold PBS and stained with anti-CXCR4, anti-CD71, anti-Ter119, and Hoechst for 20 min at 4°C. Cells were analyzed with either an LSRFortessa flow cytometer (BD Biosciences) or a Symphony A3 flow cytometer (BD Biosciences), and data were analyzed with Kaluza software (Beckman Coulter).

### Imaging flow cytometry

An Imagestream X mkII fitted with three lasers (405-, 488-, and 633-nm excitation wavelengths; Amnis, Luminex Corporation) was used for acquisition performed at either ×40 or ×60 magnification and a low-speed high-sensitivity setting with a minimum of 5000 events recorded. The gating strategy involved selecting single cells by their area and aspect ratios, which was followed by selecting focused cells on the basis of the “gradient RMS” feature. This enabled populations of proerythroblasts, early erythroblasts, late erythroblasts, pyrenocytes, reticulocytes, and erythrocytes to be distinguished from each other. The erythroblast-related parameters of MFIs, aspect ratios, delta-centroid CD71/Hoechst, bright detail similarity CXCL12/Hoechst, and Hoechst area were recorded for samples treated with or without 100 nM CXCL12-AF647 and with or without a 10-min pretreatment with 10 μM AMD3100 or pertussis toxin (0.1 μg/ml) for 45 min at 37°C. A negative correlation between erythroblast differentiation state, as defined by Ter119 MFI and co-localization of internalized CXCL12-AF647 and the nucleus (Hoechst), was analyzed by linear regression. Co-localization of CXCL12-AF647 and Hoechst was compared between the 14% of erythroblasts expressing the highest Ter119 (MFI > 20,000) and the 14% of erythroblasts expressing the lowest Ter119 (MFI < 7000). Fixation and permeabilization were performed only for the detection of intracellular CXCR4 phosphorylation with the FOXP3 Fix/Perm Buffer Set (BioLegend, 421401) according to the manufacturer’s instructions and anti–pS346 CXCR4 antibody (7TM antibodies). IDEAS software version 6.2 was used for all offline analysis. Representative events were selected manually.

### Immunofluorescence staining for confocal microscopy and image analysis

Glass-bottom Cellview cell culture dishes (Greiner Bio-One) were coated with poly-l-lysine (Sigma-Aldrich, P4707) at 37°C for at least 1 hour and washed with PBS. BM Ter119^+^ erythroid cells were isolated by magnetic cell sorting (Miltenyi Biotec) and treated with 100 nM CXCL12 for 45 min with or without a 10-min pretreatment with either 10 μM AMD3100 or pertussis toxin (0.1 μg/ml; Invitrogen, PHZ1174). Cell surface and Hoechst stainings were performed simultaneously. Fixation, permeabilization, and intracellular staining were performed with the FOXP3 Fix/Perm Buffer Set (BioLegend, 421403) according to the manufacturer’s instructions. Endogenous intracellular CXCL12 was analyzed in freshly isolated Ter119^+^ BM cells with anti-CXCL12 antibody. Cells were imaged with an inverted LSM 800 laser scanning confocal microscope (Zeiss) equipped with solid-state laser diodes (405-, 488-, 561-, and 640-nm excitation wavelengths). Serial *z* stacks were acquired with oil immersion 40×/1.3 or 63×/1.4 objectives lenses with a 0.2- to 0.5-μm step size. Acquisition was performed with ZEN software with a 1024 × 1024-pixel resolution, average of two to four frames, and 1× to 3× zoom. Images were deconvoluted with Huygens software (Scientific Volume Imaging) and analyzed with ImageJ software.

### Live-cell imaging

Glass-bottom Cellview cell culture dishes (Greiner Bio-One, 627871) were coated with poly-l-lysine (P4707, Sigma-Aldrich) at 37°C for 1 hour and washed with PBS. BM Ter119^+^ erythroid cells were isolated by magnetic cell sorting (Miltenyi Biotec). The isolated cells were allowed to adhere to the dish in IMDM medium containing cell-surface staining antibodies and Hoechst for 60 min at 37°C. Where indicated, Fluo-4 AM (Invitrogen, F10489) was added to the cells for this period. Medium was replaced by fresh IMDM containing 10% fetal calf serum (FCS), and the dishes were placed under the confocal microscope on a heated stage set at 37°C and 5% CO_2_. For live-cell CXCL12-AF647 binding and internalization, 100 nM CXCL12-AF647 was added to the dish after the live imaging commenced. The zero time point was defined as the first moment during the live imaging when CXCL12-AF647 fluorescence became apparent in the medium within the field of view under the microscope. Such a protocol was necessary because CXCL12-AF647 was gently pipetted laterally to the slide so as not to dislodge the cells and was allowed to diffuse. To reach detectable amounts during live-cell imaging, the fluorophore needed to accumulate within the field of view under the microscope because laser settings were chosen at the lower range to not oversaturate the AF647 fluorescence toward the end of the observation. Thus, the zero time point in these experiments was delayed by the period required for CXCL12-AF647 diffusion and saturation of the fluorescence and varied in different experiments. Single confocal plane images (1024 × 1024 pixels) were taken every 60 or 70 s for 60 min using a single line and frame average. For short-term Ca^2+^ flux recordings, single confocal plane images (512 × 512 pixels) were taken every 0.32 s for 1 min. In some experiments, cells were fixed after the live observation period in 4% buffered paraformaldehyde, and *z*-stack series were recorded as described earlier. Images were deconvoluted with Huygens software and analyzed with ImageJ software. For three-dimensional (3D) representations of internalized CXCL12-AF647 and CXCR4, images were processed with Velocity software (Quorum Technologies Inc.).

### Correlative fluorescence and electron microscopy

BM Ter119^+^ erythroid cells were isolated by magnetic cell sorting (Miltenyi Biotec) and were adhered to poly-l-lysine–coated, photoetched, alphanumeric gridded, coverslip-bottomed dishes (MatTek). Cells were stained with rat anti–CXCR4-AF488, anti-rat horseradish peroxidase, and Hoechst for 1 hour at 37°C while adhering before being pulsed with 100 nM CXCL12 to stimulate CXCR4 internalization. One hour after the addition of CXCL12, the cells were fixed with 2% formaldehyde and 1.5% glutaraldehyde in PBS. Erythroblasts were identified and imaged by confocal fluorescence microscopy as described earlier. Their location relative to the alphanumeric grid was recorded. The DAB reaction was performed as previously described (64). Serial ultrathin (70-nm) sections of cells, previously identified by fluorescence and light microscopy, were prepared with a diamond knife (Diatome) on an ultramicrotome (Leica) and collected either onto formvar-coated slot grids or onto indium tin oxide–coated coverslips (Diamond Coatings UK). Sections were stained with lead citrate and either imaged in a 120-kV transmission electron microscope (Tecnai Spirit Biotwin, Thermo Fisher Scientific) as previously described (65) or in a scanning electron microscope at a 4.5-kV accelerating voltage with a 3-kV stage bias, using a backscatter electron detector (Gemini 300 FEG scanning electron microscope, tandem decel and Sense BSD, Zeiss). Serial images were aligned with Fiji (66) and TrakEM2 (67). Correlation of immunofluorescence confocal microscopy images with electron microscopy images was performed manually by comparing visual landmarks of electron-dense, DAB-reactive areas with CXCR4-AF488 staining, as well as heterochromatin with Hoechst staining with Fiji and VistaCreate.

### Transwell cell migration assay

The bottom wells of a Transwell system were filled with 0, 10, 100, or 1000 nM murine CXCL12 in IMDM and 10% FCS. Transwell inserts (8-μm) were placed on top, and BM cell suspensions containing 2 × 10^6^ nucleated cells in IMDM and 10% FCS with or without 10 μM AMD3100 were added into them. The Transwells were incubated for 3 hours at 37°C and 5% CO_2_. Cells that migrated into the lower well were recovered; stained with antibodies against CD71, Ter119, and CD45, as well as with Hoechst; and were counted by flow cytometry with CountBright Absolute Counting Beads, as described earlier.

### In vitro enucleation assay

Nucleated BM cells were prepared by density centrifugation at 1000*g* for 15 min at room temperature with Lympholyte M (Cedarlane). Nucleated erythroid cells were isolated by anti-Ter119 magnetic cell sorting (Miltenyi Biotec) and resuspended at 2 × 10^6^ × 10^6^/ml to 4 × 10^6^/ml in IMDM, 15% fetal bovine serum (FBS), 1% antibiotic antimycotic solution (Sigma-Aldrich, A5955), Holo-Transferrin (300 μg/ml; Sigma-Aldrich, T1283), EPO (20 μg/ml; BioLegend, 587602), and 0.14% β-mercaptoethanol and were cultured with or without 100 nM CXCL12 and 10 μM AMD3100 overnight. The cells were then resuspended and analyzed by imaging flow cytometry as described earlier. To enable the comparison of data acquired from multiple experiments, enucleation was expressed for each group as ratios of nucleated erythroblasts and pyrenocytes and was also controlled by calculating the ratios of erythrocytes and pyrenocytes.

### In vitro enucleation assays with in vitro depletion of CXCR4

BM cells from CXCR4-deficient Cxcr4^fl/fl^ Ubc-creERT2 mice and their CXCR4-sufficient Cxcr4^fl/fl^ control littermates were depleted of CD11b^+^, CD5^+^, B220^+^, and Ly6G^+^ cells by magnetic cell sorting (Miltenyi Biotec) and cultured at 2 × 10^6^/ml to 4 × 10^6^/ml for 2 days with 100 nM CXCL12, with or without 25 nM (*Z*)-4-hydroxytamoxifen (Sigma-Aldrich, H7904) in IMDM, 15% FBS, Holo-Transferrin (300 μg/ml; Sigma-Aldrich, T1283), EPO (20 μg/ml; BioLegend, 587602), insulin-like growth factor 1 (5 μg/ml; R&D Systems, 791-MG-050), SCF (50 μg/ml; BioLegend, 579702), granulocyte colony-stimulating factor (50 μg/ml; Peprotech 250–05), and granulocyte-macrophage colony-stimulating factor (20 μg/ml; Peprotech, 315-03). Cells were harvested, staining of erythroid cells was performed as described earlier, and cells were acquired using CountBright Absolute Counting Beads (Life Technologies) on a Symphony A3 flow cytometer (BD Biosciences) and analyzed by Kaluza software (Beckman Coulter). Data are expressed as ratios of (*Z*)-4-hydroxytamoxifen–treated and vehicle-treated samples for erythrocytes or pyrenocytes of both genotypes.

### In vivo CXCR4 antagonist treatment

Mice were injected with LY2510924 (5 mg/kg, subcutaneous) or 100 μl of PBS. Three hours after injection, BM was harvested from femora, tibiae, and humeri by flushing with PBS. Cells were passed through a 70-μm strainer and washed with PBS. Cells were resuspended in PBS and 0.5% bovine serum albumin (BSA) and stained with antibodies against CD71, Ter119, and CD45, as well as with Hoechst 33342. Erythroid cells at different stages of erythropoiesis were defined as CD45^−^ and CD71^hi^Ter119^int^Hoechst^int^ (I, proerythroblasts), CD71^hi^Ter119^hi^Hoechst^int^Fschi (II, early erythroblasts), or CD71hiTer119hiHoechstintFscint (III, late erythroblasts). The gating strategy used is shown in fig. S1E.

### Erythroblast isolation and extraction for kinome analysis

Nucleated BM cells were prepared by density centrifugation at 1000*g* for 15 min at room temperature with Lympholyte M. Nucleated erythroid cells were isolated by anti-Ter119 magnetic cell sorting (Miltenyi Biotec) and resuspended in IMDM with or without 100 nM CXCL12 and incubated for 10 or 60 min at 37°C. The cells were then washed with ice-cold PBS and pelleted at 500*g* for 8 min at 4°C and stored at −80°C until shipment for analysis.

### Serine-threonine kinase activity profiling with PamChip peptide microarrays

The mapping of serine-threonine (Ser/Thr) kinase activities in erythroblasts was performed by Pamgene (‘s-Hertogenbosch, The Netherlands) as described previously (68). The Ser/Thr profiles were measured with the PamChip peptide STK flow-through microarray system on PamStation12. The STK PamChip Array contained four positive control peptides and 140 serine/threonine peptides. The peptides immobilized on the PamChip consisted of 15–amino acid sequences and corresponded to putative phosphorylation sites, which served as Ser/ Thr kinase substrates. The phosphorylation activities of Ser/Thr kinases were visualized with a two- step assay method including a mixture of primary antibodies and a fluorescently (FITC-) labeled secondary antibody. Erythroblast cell pellets were lysed in M-PER Mammalian Protein Extraction Reagent (Thermo Fisher Scientific, no. 78503), and 1 μg of total erythroid cell protein was used for Ser/Thr kinase activity profiling according to the standard protocol. All reagents used for the preparation of the Ser/Thr Basic Mix and the Detection Mix were supplied by Pamgene International B.V. The STK Basic Mix was composed of the protein lysate, 4 μl of 10× PK, 0.4 μl of 100× BSA, 4.0 μl of 4 mM adenosine 5′-triphosphate, and 0.46 μl of STK antibody mix. The total volume of the STK Basic Mix was adjusted to 40 μl by adding 27 μl of distilled water. The detection mix (total volume, 30 μl) consisted of 3 μl of 10× antibody buffer, 0.4 μl of FITC-labeled STK antibody, and 26.6 μl of distilled water. After performing PamChip preprocessing from cycles 1 to 30, the STK Basic Mix was applied to each of the four arrays of the three PamChips. The PamChip microarrays were then incubated and washed for 60 cycles (cycles 30 to 90). In a second step, the Detection Mix was transferred to the PamChips. Incubation with FITC-labeled STK antibody and image recording (after 10, 50, and 200 ms) were performed for the next subsequent 30 cycles (starting with cycle 92), and an end- level read was performed after the final cycle 124 (10, 20, 50, 100, and 200 ms). One outlier (UT12) due to insufficient antibody binding was excluded to ensure valid analysis.

### Upstream kinase analysis

Upstream kinase analysis was based on Ser/Thr kinase phosphorylation patterns and automatically calculated by default with the PamApp on BioNavigator Analysis software tool (Pamgene). Peptides spotted on the chip were either selected by their ability to be phosphorylated by multiple kinases or by their recognition only by distinct kinases. The collection of peptides provides substrates for the whole kinome and enables full coverage for all kinases with annotated downstream substrate proteins known so far. Prediction of the differentially activated upstream kinases was performed by a comparison of the individually determined specific pattern of substrate peptide phosphorylation for experimental groups, CXCR4-sufficient versus CXCR4-deficient without any treatment as well as CXCR4-sufficient and CXCR4-deficient treated with 100 nM CXCL12 for 10 min versus 60 min were analyzed, with databases containing empirical in vitro/in vivo (iviv) as well as literature-based protein modifications, such as HPRD, PhosphoELM, PhosphositePLUS, Reactome, and UniProt, and an in silico predictions database (PhosphoNet; www.phosphonet.ca/). Kinases predicted by the iviv databases were given higher weightage (rank = 0). Rankings according to PhosphoNET (from 1 to 12) were sorted by their proprietary algorithms or by modeling based on kinase active sites and predictive values. The normalized median kinase statistics depicts the overall change of the peptide set that represents a substrate group for a given protein kinase. The specificity score (*Q*sp) indicates the specificity of the normalized kinase statistics with respect to the number of peptides used for predicting the corresponding kinase. The greater the Qsp, the less likely it is that the normalized kinase statistic could have been generated using a random set of peptides from the present dataset. *Q*sp logarithmic values > 1.3 were considered as statistically relevant. The final ranking of the kinases was based on median final score (*Q*), which was calculated by addition of the significance score and the specificity score (*Q* = *Q*sg + *Q*sp). To visualize the Ser/Thr profiling data and functional relationships, a phylogenetic tree was generated with the web-based kinome tool KinMAPbeta (http://kinhub.org/kinmap/) and the CORAL tool (http://phanstiel-lab.med.unc.edu/CORAL/).

### Statistical analysis

Data analysis was performed with Prism 9 software (GraphPad). Differences between two groups were assessed for statistical significance using two-tailed paired/unpaired Student’s *t* tests. One-way or two-way analysis of variance (ANOVA) with Tukey, Dunnett, or Holm Šidák post hoc tests was performed for multiple group comparisons as appropriate. Results were considered significantly different when *P* < 0.05, with values designated as follows: *P < 0.05, **P < 0.01, ***P < 0.005, and ****P < 0.001. All data are expressed as means ± SEM, and *n* numbers and the statistical tests used for each dataset are detailed in the figure legends. Data were excluded in case of significant outlier test.

## Supplementary Material

Supplementary check list

Supplementary data file

Supplementary File

Supplementary Movies

## Data Availability

scRNA-seq data are deposited at GEO under accession number GSE272318. All data needed to evaluate the conclusions in the paper are present in the paper and/ or the Supplementary Materials.

## Teaser

CXCL12-stimulated CXCR4 signaling mediates erythroblast enucleation to generate red blood cells in mice.

## Editor’s summary

Signaling by the chemokine CXCL12 through its receptor CXCR4 promotes immune cell migration during embryonic development, immune surveillance and to sites of infection and inflammation. Gutjahr *et al*. found that bone marrow–derived CXCL12 activated CXCR4 on mouse bone marrow erythroblasts, the precursors of red blood cells (erythrocytes), to promote erythroblast differentiation, rather than their migration, through pathways involving intracellular and nuclear CXCR4 signaling. Knockout of CXCR4 specifically in erythroblasts inhibited erythrocyte generation in mice. These findings indicate that CXCL12-CXCR4 signaling mediates erythropoiesis, suggesting that it should be investigated for the treatment of erythrocyte pathologies. —John F. Foley
