# Supplementary material for: Intracellular and nuclear CXCR4 signaling promotes terminal erythroblast differentiation and enucleation
Source: Sci Signal. Author manuscript; Available in PMC 2026 Aug 10. (PMC7619343; doi:10.1126/scisignal.adt2678)
Supplement: Supplementary file 1 [file EMS217195-supplement-Supplementary_File.pdf]

Science Signaling
AAAS

Supplementary Materials for

Intracellular and nuclear CXCR4 signaling promotes terminal erythroblast differentiation and enucleation

Julia Christine Gutjahr *et al.*

Corresponding author: Julia Christine Gutjahr, julia.gutjahr@bitg.ch; Antal Rot, a.rot@qmul.ac.uk

Sci. Signal. **18**, eadt2678 (2025)
DOI: 10.1126/scisignal.adt2678

The PDF file includes:

Figs. S1 to S8
Table S1
Legends for movies S1 to S6
Legend for data S1
Reference (*69*)

Other Supplementary Material for this manuscript includes the following:

Movies S1 to S6
Data file S1
MDAR Reproducibility Checklist

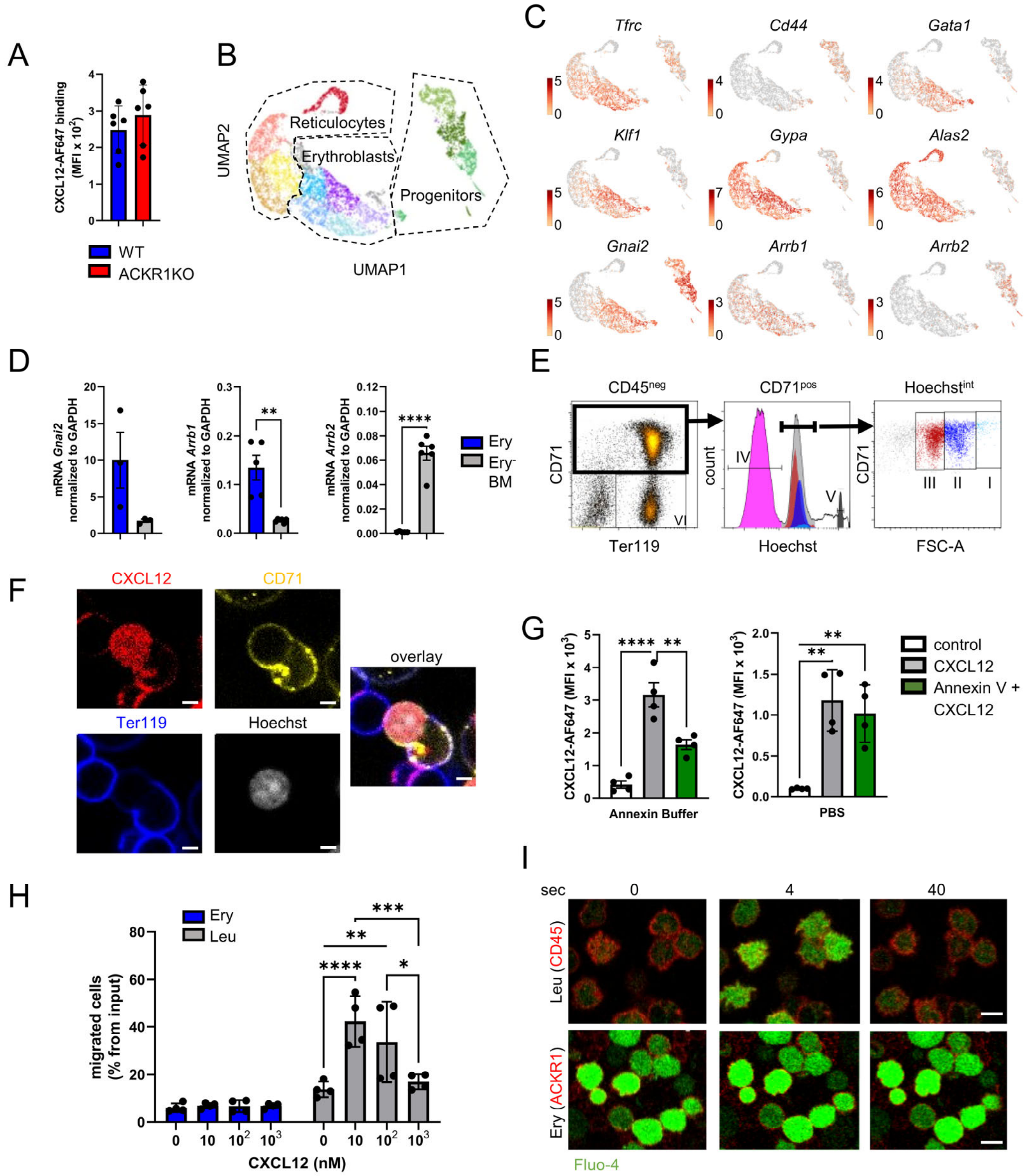

Fig. S1. Molecular makeup and CXCR4-induced responses of BM erythroid cells. (**A**) CXCL12-AF647 binding to BM CD71$^+$Ter119$^+$ erythroblasts from WT and ACKR1-knockout (KO) mice measured by flow cytometry (n = six mice; $P < 0.05$ by paired two-tailed t-test). (**B**) UMAP projections of scRNA-seq data of CD71$^+$ BM cells from a WT mouse identified multiple clusters, grouped as follows: progenitors (*Tfrc*$^+$, *Cd44*$^+$), erythroblasts (*Tfrc*^{high}, *Gata1*^{high}, *Klf1*^{high}), and reticulocytes *(Gypa*^{high}, *Alas2*high). (**C**) Log_2 transcription of *Tfrc*, *Cd44*, *Gata1*, *Klf1*, *Gypa*, *Alas2*, *Gnai2*, *Arrb1*, and *Arrb2* from scRNA-seq data of CD71$^+$ BM cells. (**D**) Relative abundances of *Gnai2*, *Arrb1*, and *Arrb2* transcripts in Ter119$^+$ erythroid cells (Ery) and Ery-depleted BM cells (Ery$^-$ BM) measured by qPCR and normalized to *GAPDH* abundance. (*Gnai2*: n = three mice; *Arrb1* and *Arrb2*: n = five mice; $**P < 0.01$, $****P < 0.001$ by unpaired two-tailed t test). (**E**) Flow cytometry gating strategy used to define CD45$^-$ erythroid subpopulations in murine BM cells: proerythroblasts (I), early erythroblasts (II), late erythroblasts (III), reticulocytes (IV), pyrenocytes (V), and erythrocytes (VI). (**F**) CXCL12-AF647 binding to a pyrenocyte as determined by confocal microscopy (CXCL12: red; CD71: yellow; Ter119: blue; nucleus, Hoechst: white) Scale bars, 2 µm. (**G**) CXCL12-AF647 binding to BM pyrenocytes with and without Annexin V as measured by flow cytometry

in Annexin buffer (left) and in PBS (right). Data are from n = four mice. $**P < 0.01$, $****P < 0.001$ by one-way ANOVA with Tukey's multiple comparisons test. (**H**) CXCL12-induced migration of $CD71^+Ter119^+$ erythroblasts (Ery) and leukocytes (Leu) after 3 hours in a Transwell assay. Data are from n = four mice. $*P < 0.05$, $**P < 0.01$, $****P < 0.001$ by two-way ANOVA with Tukey's multiple comparisons test. (**I**) Representative images from movies S1 and S2 showing free intracellular Ca^{2+} detected after stimulation with CXCL12 as determined by Fluo-4 fluorescence (green). Top: leukocytes, $CD45^+$ (red); Bottom: Erythroid cells, ACKR1 (red) at 0 (left), 4 (middle), and 40 s (right) after treatment. Scale bars, 10 µm. Data are means ± SEM. Each data point represents a single mouse.

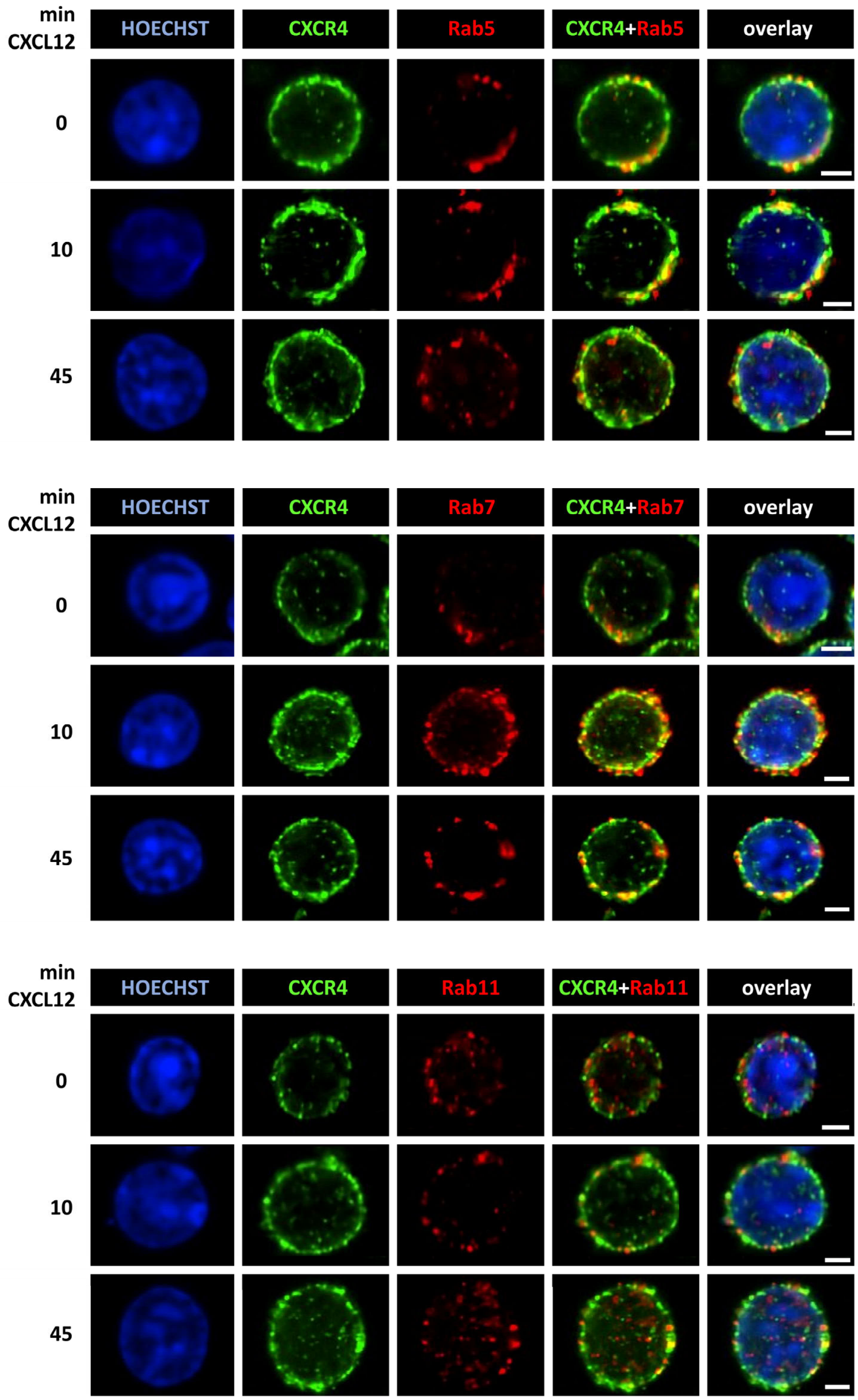

Fig. S2. CXCR4 co-localization with endosomal markers. Confocal microscopy images of freshly isolated murine BM erythroblasts that were stimulated ex vivo for 0, 10, or 45 min with CXCL12, fixed and permeabilized, and then stained with anti-CXCR4 antibody (green), anti-Rab antibodies, as indicated, (red) and Hoechst (blue). Scale bars, 2 µm. Images are representative of three experiments.

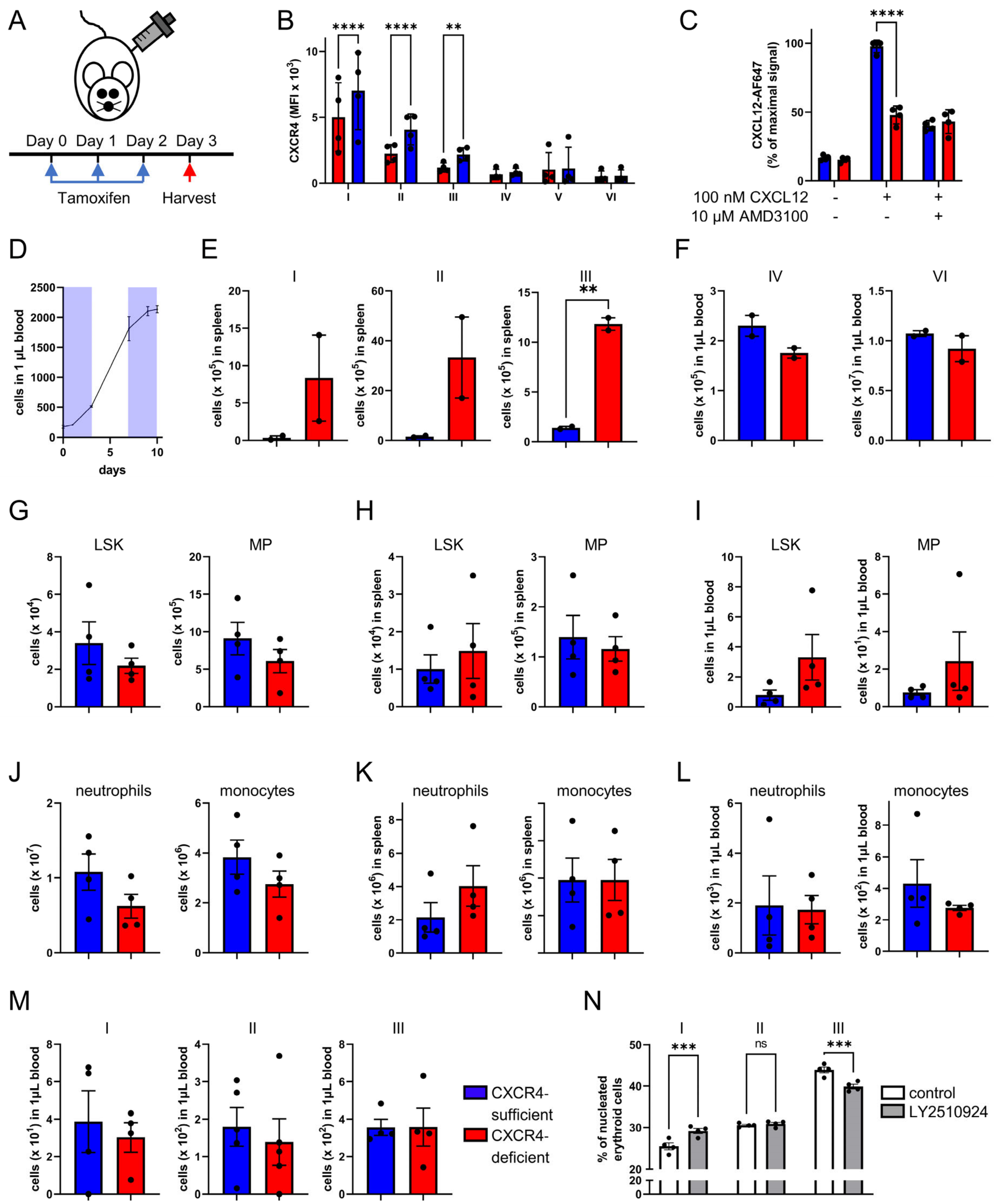

Fig. S3. CXCR4 depletion and cell counts in a *CXCR4*-floxed UBC-ERT2-Cre mouse model. (**A**) Schematic representation of the in vivo tamoxifen treatment protocol of *CXCR4*-floxed UBC-ERT2 Cre (CXCR4-deficient) and *CXCR4*-floxed (CXCR4-sufficient) mice. (**B**) Anti-CXCR4 antibody reactivity with murine BM proerythroblasts (I), early erythroblasts (II), late erythroblasts (III), reticulocytes (IV), pyrenocytes (V), and erythrocytes (VI) from CXCR4-sufficient (blue) and -deficient (red) mice as measured by flow cytometry. Data are from four mice per group. $^{**}P < 0.01$, $^{****}P < 0.001$ by two-way ANOVA with Šídák's multiple comparisons test. (**C**) CXCL12-AF647 binding and its inhibition by the CXCR4

antagonist AMD3100 in early erythroblasts from CXCR4- sufficient and -deficient mice as measured by flow cytometry. Data are from four mice per group. ****$P < 0.001$ by two-way ANOVA with Šídák's multiple comparisons test. (**D**) Mobilization of nucleated CD71$^+$ erythroblasts into the blood of CXCR4-deficient mice after two cycles of three tamoxifen injections on three consecutive days (blue). (**E** and **F**) Total numbers of proerythroblasts (I), early erythroblasts (II), and late erythroblasts (III) in whole spleen (E) and total numbers of reticulocytes (IV) and erythrocytes (VI) in 1 µl of blood (F) in CXCR4-sufficient and -deficient mice after two cycles of three tamoxifen injections on three consecutive days. Data are from two mice per genotype and were analyzed by unpaired two-tailed *t* tests. (**G** to **I**) Total numbers of Lineage$^-$ Sca1$^+$c-kit$^+$ cells (LSKs) and myeloid progenitors (MPs) in bone marrow (from six bones) (G), whole spleen (H), and 1 µl of blood (I) in CXCR4-sufficient and -deficient mice. (**J** to **L**) Total numbers of neutrophils and monocytes in bone marrow (from six bones) (J), whole spleens (K), and 1 µl of blood (L) in CXCR4-sufficient and -deficient mice. (**M**) Total numbers of proerythroblasts (I), early erythroblasts (II), and late erythroblasts (III) in 1 µl of blood from CXCR4- sufficient and -deficient mice. (G to M) Data are from four mice per genotype and were analyzed by unpaired two-tailed *t* tests. (**N**) Percentages of BM CD71^{hi}Ter119^{int}Hoechst^{int} (I, proerythroblasts), CD71^{hi}Ter119^{hi}Hoechst^{int}Fsc^{hi} (II, early erythroblasts), and CD71^{hi}Ter119^{hi}Hoechst^{int}Fsc^{int} (III, late erythroblasts) in total BM erythroid cells three hours after the mice were injected with the CXCR4 antagonist LY2510924 or vehicle as a control. Data are from four mice per group. ****$P < 0.001$ by two-way ANOVA with Šídák's multiple comparisons test.

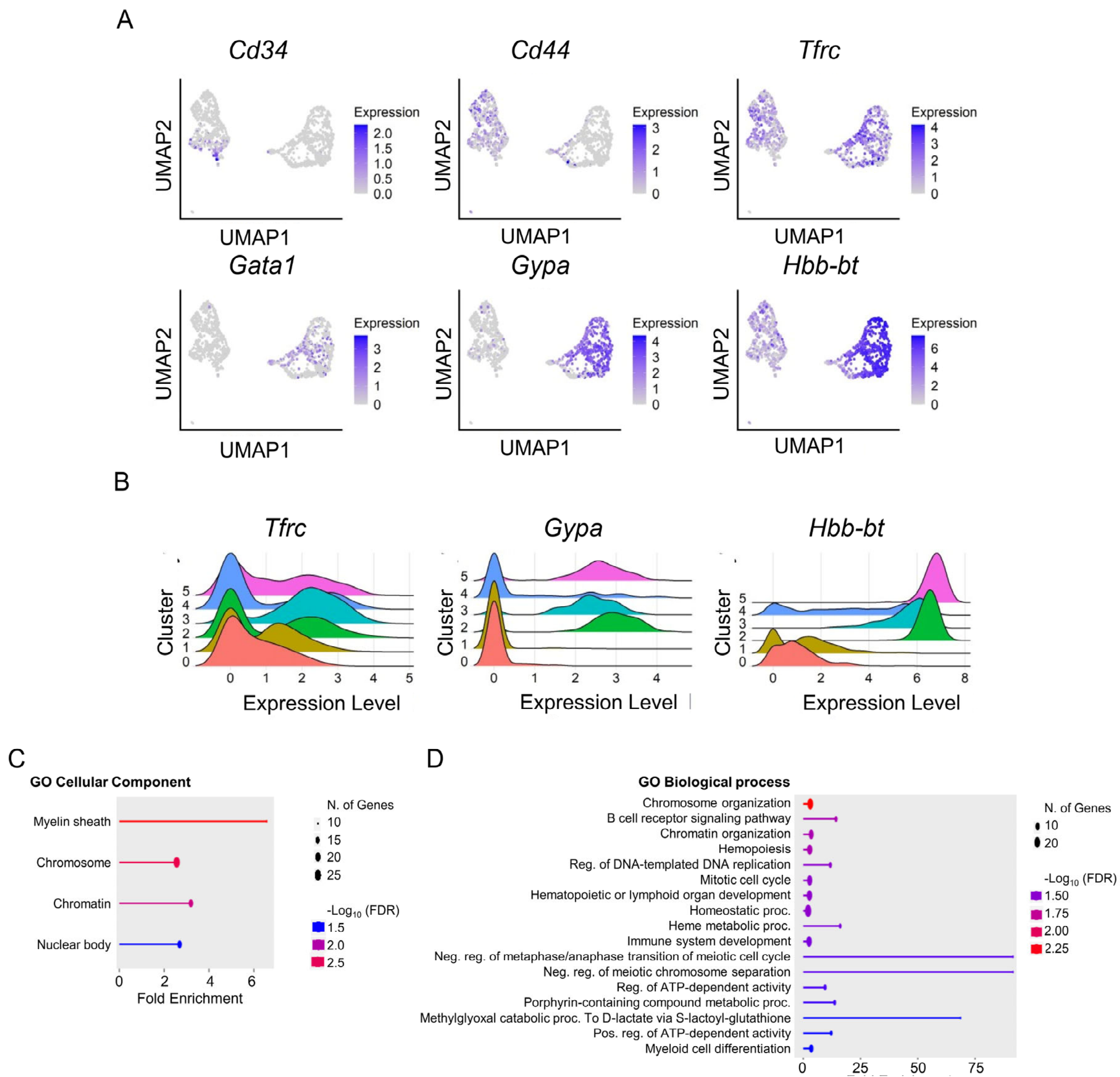

Fig S4. Erythroid cell clustering and differentially expressed genes in CXCR4-sufficient versus CXCR4-deficient erythroblasts. (**A**) Log_2 values for the transcription of *Cd34*, *Cd44*, *Tfrc*, *Gata1*, *Gypa*, and *Hbb-bt* from scRNA-seq analysis of $CD71^+$ BM cells. UMAP projections of combined data from scRNA-seq analysis of CXCR4-sufficient and -deficient $CD71^+$ BM cells. (**B**) Log_2 values for the transcription of *Tfrc*, *Gypa*, and *Hbb-bt* in the five identified clusters, grouped as progenitors (clusters 0 to 1) and erythroblasts (clusters 2 to 5) according to pseudotime analysis and gene expression. (**C**) GO (Cellular Component) analysis of genes decreased in expression in CXCR4-deficient $CD71^+$ BM cells. Log_2 of the fold-change < -0.5. (**D**) GO (Biological Process) analysis of genes increased in expression in CXCR4-deficient $CD71^+$ BM cells. Log_2 of the fold-change > 0.5.

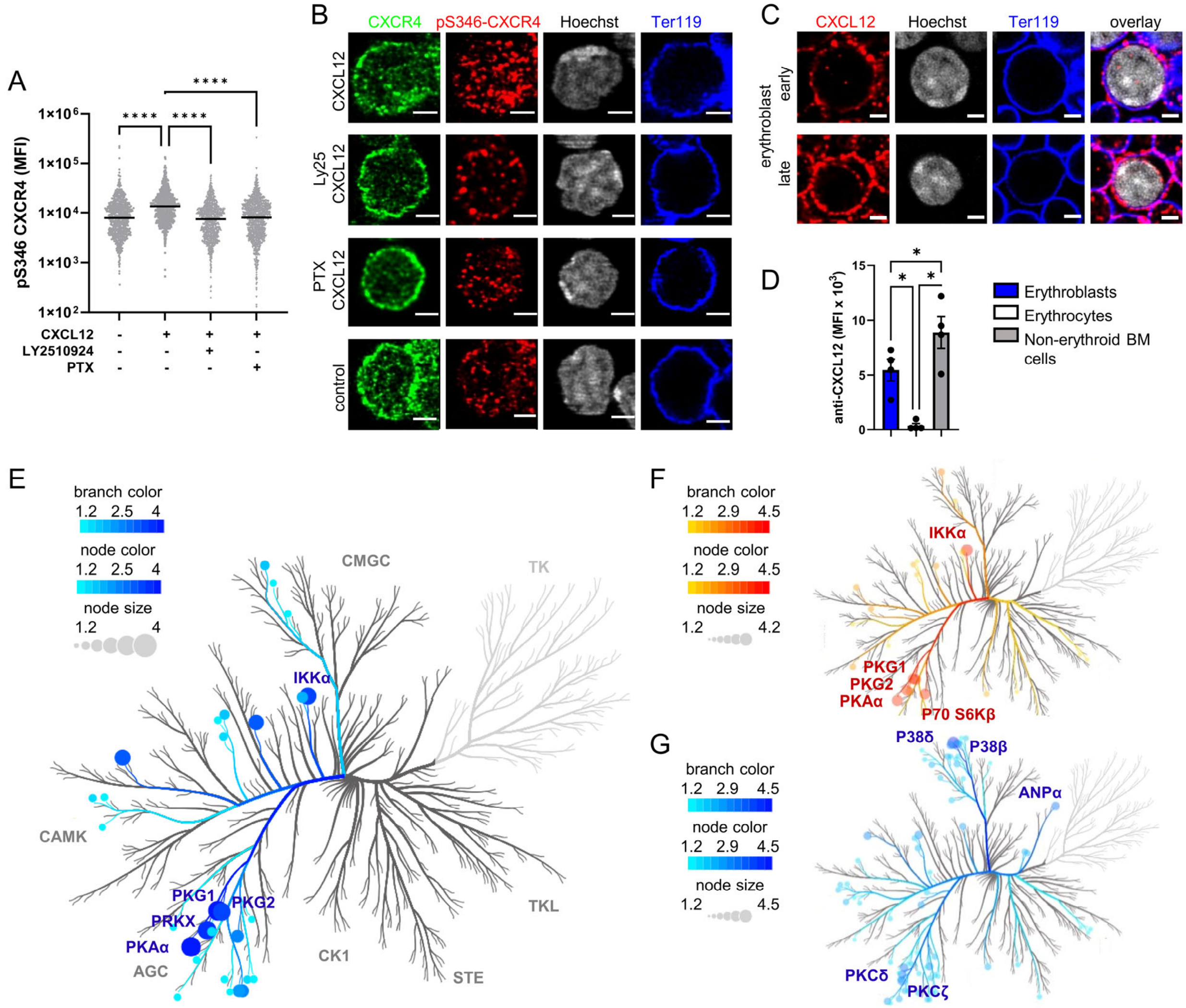

Fig. S5. CXCL12-induced phosphorylation of CXCR4 and its nuclear localization in erythroblasts. (**A**) Mean fluorescence intensity (MFI) values of anti-pS346-CXCR4 antibody staining of freshly isolated CD71$^+$Ter119$^+$ BM erythroblasts stimulated with CXCL12 in the presence or absence of the CXCR4-antagonist LY2510824 or pertussis toxin (PTX) and analyzed by imaging flow cytometry. Individual and median values for 655 to 1193 cells per group are shown for one experiment, which is representative of three experiments. ****P < 0.001 by one-way ANOVA with Tukey's multiple comparisons test. (**B**) Confocal images of 0.2 µm optical sections of freshly isolated murine BM erythroblasts stimulated with CXCL12 and stained with anti-CXCR4 antibody (green), anti-pS346 CXCR4 antibody (red), anti-Ter119 antibody (blue), and Hoechst (white) with or without pre-treatment with either LY2510824 or PTX. Scale bars, 5 µm. (**C**) Anti-CXCL12 antibody reactivity (red) in untreated freshly isolated Ter119$^+$ (blue) early (top) and late (bottom) BM erythroblasts as categorized by the size and density of their nuclei. Scale bars, 5 µm. (**D**) Endogenous cell-associated CXCL12 quantified in the indicated BM cell populations by flow cytometry with anti-CXCL12 antibody. Data are means ± SEM of four mice. *P < 0.05 by one-way ANOVA with Tukey's multiple comparisons test. (**E**) Coral-tree representation of upstream kinase analysis (UKA) of serine-threonine kinase activities according to their median scores in CXCR4-deficient erythroblasts isolated from the bone marrow of Cxcr4$^{fl/fl}$Ubc-creERT2 mice as compared to CXCR4-

sufficient erythroblasts isolated from control littermates. The gradient of blue in the coral tree branches indicates decreased activity. The top twelve hits of downregulated kinases are labelled in blue (see data file S1). (**F**) Coral-tree representation of serine-threonine kinase activities in CXCR4-sufficient erythroblasts after 60 min of CXCL12 treatment compared to those after 10 min of treatment. The gradient of red in the coral tree branches indicates increased activity. The top five hits of upregulated kinases are labelled in red (see data file S1). (**G**) Coral-tree representation of serine-threonine kinase activities in CXCR4-deficient erythroblasts after 60 min of CXCL12 treatment compared to those after 10 min of treatment. The gradient of blue in the coral tree branches indicates decreased activity. The top five hits of downregulated kinases are labelled in blue (see data file S1). (**E** to **G**) Kinase families are labelled in gray as follows. CMGC: CDKs (cyclin-dependent kinases), MAPKs (mitogen-activated protein kinases), GSKs (glycogen synthase kinases), and CDK-like kinases; CAMK: $Ca^{2+/}$calmodulin-dependent protein kinases; AGC (cAMP-dependent, cGMP-dependent, and protein kinase C); casein kinase 1 (CK1); STE (homologs of yeast sterile 7, Sterile 11, and sterile 20 kinases); TKLs: tyrosine kinase–like kinases; TKs: tyrosine kinases. Analysis was performed on erythroblasts from five mice of each group with branch color, node color, and node size reflecting the median final scores for the individual kinases. Connecting branches reflect kinase relationships.

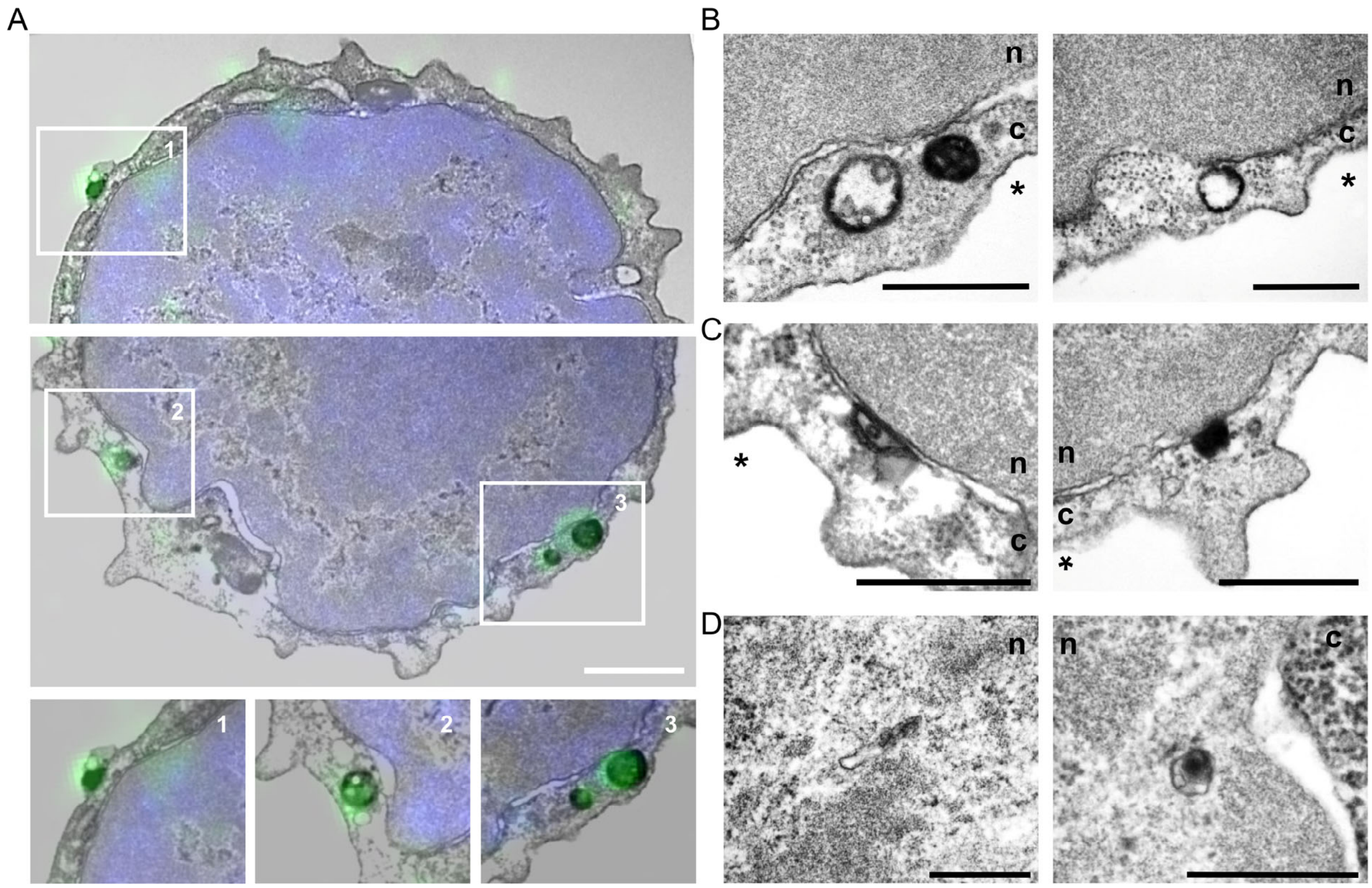

Fig. S6. Subcellular localization of CXCR4 in erythroblasts as determined by correlative fluorescence and immune-electron microscopy. (**A**) Subcellular localization of two CXCR4-associated immune labels in an erythroblast observed by correlative confocal immunofluorescence microscopy and by transmission immune-electron microscopy. The image is representative of eight cells. The image shows two segments of an erythroblast at different z-depths. Freshly isolated viable erythroblasts were stimulated with CXCL12, pulsed with rat anti–CXCR4-AF488 (green) and a horseradish peroxidase (HRP)–labeled goat anti-rat Ig antibody and counterstained with Hoechst (blue). The fluorescent signal was detected in 0.3-µm optical confocal microscopy sections. The HRP was visualized by its substrate 3,3-diaminobenzidine (DAB), which was catalyzed to form an electron-dense precipitate detected in ultrathin sections by transmission electron microscopy. The confocal and ultrastructural images were digitally aligned. Scale bar, 1 µm. Separated fluorescence and electron microscopy images are shown in fig. S7. Area 1 shows CXCR4 endocytosis from the membrane. Areas 2 and 3 show $CXCR4^{+}$ large multivesicular bodies in close proximity and associated with the nuclear membrane. (**B** to **D**) CXCR4-associated signal in erythroblasts detected by transmission electron microscopy as an electron-dense DAB precipitate in (B) multivesicular bodies associated with the nuclear envelope; (C) on the outer layer of the nuclear envelope, and (D) in the nucleoplasmic reticulum. n, nucleus; c, cytoplasm; an asterisk indicates the extracellular space. Scale bars, 0.5 µm.

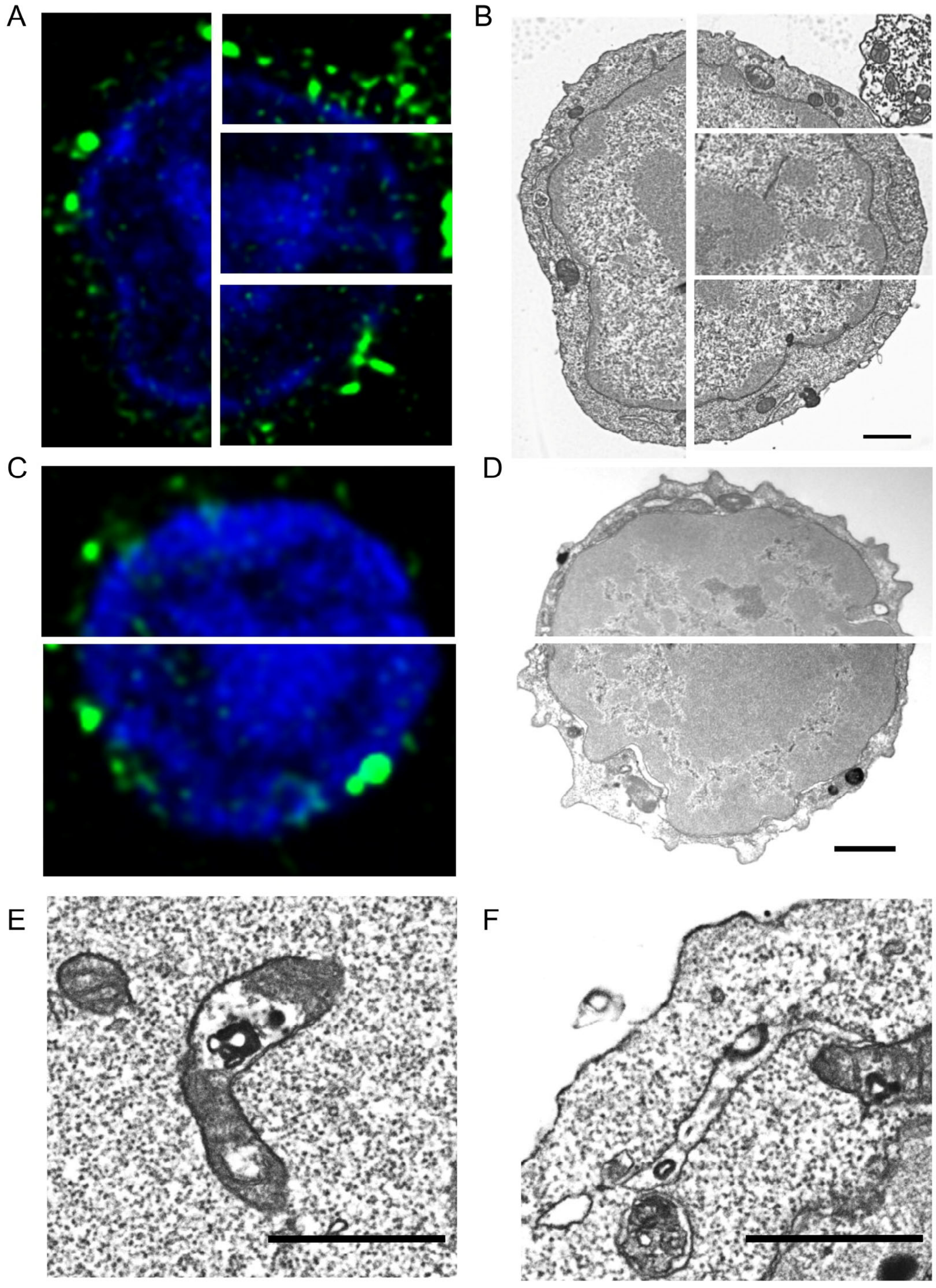

Fig. S7. Side-by-side image separation of correlative fluorescence and electron microscopy and immune-electron microscopy with subcellular localization of CXCR4 in erythroblasts. (**A** and **B**) These images correspond to those shown in Fig. 4F. (A) Confocal immunofluorescence microscopy shows anti-CXCR4-AF488 (green) immunoreactivity and Hoechst DNA staining (blue) in an erythroblast with four segments of the same cell at different z-depths. The fluorescent signal was detected in 0.3-μm optical confocal microscopy sections. (B) Electron microscopy images of the same erythroblast as that shown in (A) at a corresponding z-depth. Scale bar, 1 μm. (**C** and **D**) These images correspond to those shown in fig.

S6A. (C) Confocal immunofluorescence microscopy image of anti-CXCR4-AF488 (green) immunoreactivity and Hoechst DNA staining (blue) in an erythroblast showing two segments of the same cell at different z-depths. The fluorescent signal was detected in 0.3-μm optical confocal microscopy sections. (**D**) Electron microscopy image of the same erythroblast as that shown in (C), with rat anti-CXCR4-AF488 and a horseradish peroxidase (HRP)-labeled goat-anti-rat Ig antibody. The HRP was visualized by its substrate DAB, which was catalyzed to form an electron-dense precipitate detected in ultrathin sections by transmission electron microscopy. Scale bar, 1 μm. (**E** and **F**) CXCR4-associated signal in erythroblasts detected by transmission electron microscopy as an electron-dense DAB precipitate in mitochondria (E) and the endoplasmic reticulum (F). Scale bars, 1 μm.

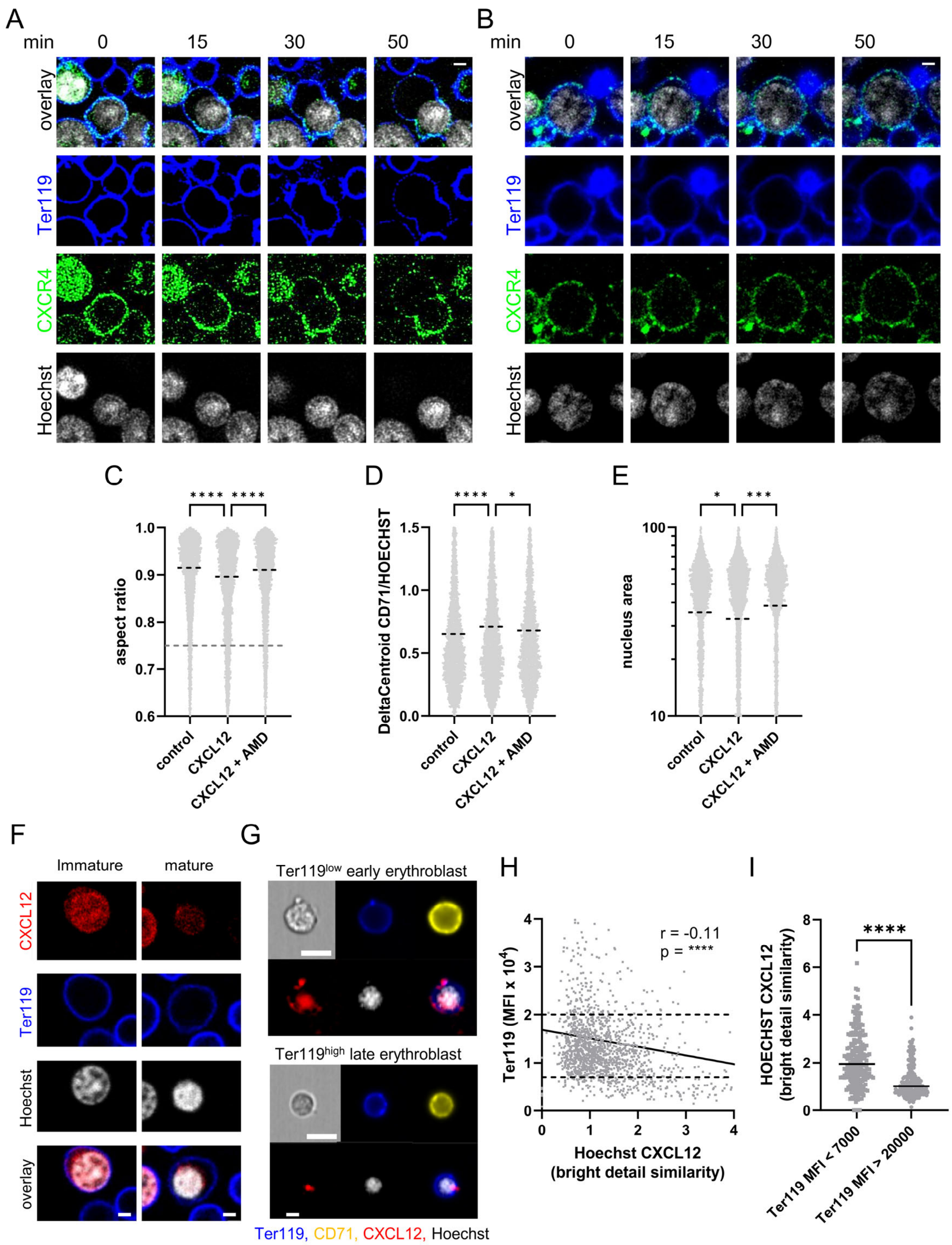

Fig. S8. CXCL12 stimulates erythroblast elongation, CXCR4 redistribution, nuclear polarization, and chromatin condensation. (**A**) Representative images from movie S5 showing BM-derived erythroblasts pre-labeled with anti-CXCR4 antibody (green), anti-Ter119 antibody (blue), and the nuclear dye Hoechst (white), which were stimulated with CXCL12 during imaging. Scale bar, 2 µm. (**B**) Representative images from live-cell imaging of BM-derived erythroblasts pre-labeled with anti-CXCR4 antibody (green), anti-Ter119 antibody (blue), and the nuclear dye Hoechst (white) without the addition of

CXCL12. Scale bar, 2 μm. (**C** to **E**) Imaging flow cytometry analysis of BM-derived erythroblasts treated with CXCL12, alone or with AMD3100, showing calculated (C) cell aspect ratios (dotted line delineates elongated erythroblast with aspect ratios of 0.75 or lower), (D) dislocation of the nucleus from the cell center, measured as a delta-centroid of CD71 and Hoechst stains, and (E) nuclear area, measured as the size of the Hoechst stain. Cumulative results from three individual experiments with cells from a single mouse (total of 3357 and 4210 cells per group). *$P < 0.05$, ***$P < 0.005$, ****$P < 0.001$ by one-way ANOVA with Tukey's multiple comparison tests. (**F**) Confocal images illustrating CXCL12-AF647 (red) internalization by an immature erythroblast (left) and a mature erythroblast (right), as defined by the sizes and densities of their nuclei, showing Ter119 (blue) and Hoechst (white). Scale bars, 2 μm. (**G**) CXCL12-AF647 (red) internalization by early- and late-stage erythroblasts as determined by imaging flow cytometry, showing Ter119 (blue), CD71 (yellow), and Hoechst (white). Scale bars, 10 μm. (**H**) Linear regression between Ter119 MFI and Hoechst-CXCL12 bright detail similarity as measured by imaging flow cytometry. Dotted lines delineate the top and bottom 14% of $Ter119^+$ 1563 cells from a single experiment representative of three experiments. Solid line shows simple linear regression. (**I**) Median bright detail similarity of CXCL12-AF647 and Hoechst in early- and late-stage erythroblasts from 212 and 218 cells, respectively. ****$P < 0.001$ by unpaired two-tailed *t* test.

Table S1. Antibodies used in this study.

Anti-mouse ACKR1 Alexa Fluor 647 (6B7)	Thiriot *et al.*, 2017 (*69*)	6B7
Anti-mouse ACKR1 Alexa Fluor 488 (6B7)	Thiriot *et al.*, 2017 (*69*)	6B7
Anti-mouse CD184 (CXCR4) Monoclonal Antibody (2B11), Alexa Fluor 488, eBioscience	Thermo Fisher Scientific	Cat# 53-9991-80, RRID:AB_953573
Anti-mouse CD184 (CXCR4) Monoclonal Antibody (2B11), eFluor 450, eBioscience	Thermo Fisher Scientific	Cat# 48-9991-82 (also 48-9991), RRID:AB_2574143
Anti-mouse CD71 PE (RI7217)	Biolegend	Cat# 113808; RRID: AB_313569
Anti-mouse CD71 FITC (RI7217)	Biolegend	Cat# 113806; RRID: AB_313567
Anti-mouse CD71 Alexa Fluor 647 (C2F2)	BDbiosciences	Cat# 563504; RRID: AB_2738245
Anti-mouse TER-119/Erythroid Cells FITC (TER-119)	Biolegend	Cat# 116206 (also 116205), RRID:AB_313707
Anti-mouse TER-119/Erythroid Cells Brilliant Violet 421(TM) (TER-119)	Biolegend	BioLegend Cat# 116233 (also 116234), RRID:AB_10933426

Anti-mouse TER-119/Erythroid Cells Alexa Fluor(R) 488 (TER-119)	Biolegend	BioLegend Cat# 116215, RRID:AB_493402
Anti-mouse TER-119/Erythroid Cells PE/Cyanine7 (TER-119)	Biolegend	BioLegend Cat# 116222 (also 116221), RRID:AB_2281408
Anti-mouse Ter119 Alexa Fluor 647 (TER-119)	Biolegend	Cat# 116218; RRID: AB_528961
Anti-mouse TER-119 Monoclonal Antibody (TER-119), eFluor 450, eBioscience	Thermo Fisher Scientific	Cat# 48-5921-82, RRID:AB_1518808
Anti-mouse CD11b Pacific Blue (M1/70)	Biolegend	Cat# 101224; RRID: AB_755986
Anti-mouse CD45 BV711 (30-F11)	Biolegend	Cat# 103147; RRID: AB_2564383
Anti-mouse CD45 PerCP/Cyanine5.5 (30-F11)	Biolegend	Cat# 103131 (also 103132), RRID:AB_893344
Anti-mouse CD45 Alexa Fluor(R) 647 (30-F11)	Biolegend	Cat# 103124 (also 103123), RRID:AB_493533
Anti-mouse CD115 (c-fms) Monoclonal Antibody (AFS98), PE, eBioscience	Thermo Fisher Scientific	Cat# 12-1152-82, RRID:AB_465808

Anti-mouse Ly-6G APC/Cyanine7 (1A8)	Biolegend	Cat# 127623 (also 127624), RRID:AB_10645331
Anti-mouse CD150 (SLAM) PerCP/Cyanine5.5 (TC15-12F12.2)	Biolegend	Cat# 115921 (also 115922), RRID:AB_2206887
Anti-mouse CD34 Monoclonal Antibody (RAM34), Alexa Fluor 700, eBioscience	Thermo Fisher Scientific	Cat# 56-0341-82, RRID:AB_493998
Anti-Mouse CD48 BD Horizon BV510 Hamster (HM48-1	BD Biosciences	Cat# 563536, RRID:AB_2738266
Anti-mouse CD16/32 APC/Cyanine7 (93)	Biolegend	Cat# 101327 (also 101328), RRID:AB_1967102
Anti-mouse Ly-6A/E (Sca-1) Brilliant Violet 605(TM) (D7)	Biolegend	Cat# 108134 (also 108133), RRID:AB_2650926
Anti-mouse CD117 (c-kit) PE/Cyanine7 (2B8)	Biolegend	Cat# 105813 (also 105814), RRID:AB_313222
Anti-mouse B220 Pacific Blue (RA3-6B2)	Biolegend	Cat# 103227; RRID: AB_492876

Anti-mouse CD3 Pacific Blue (17A2)	Biolegend	Cat# 100214; RRID: AB_493645
Anti-mouse CD11b Pacific Blue (M1/70)	Biolegend	Cat# 101224; RRID: AB_755986
Anti-mouse Gr1 Pacific Blue (RB6-8C5)	Biolegend	Cat# 108430; RRID: AB_893556
SDF1 alpha	Abcam	Cat# ab25117, RRID:AB_2088164
Recombinant Alexa Fluor 555 Anti-Lamin A and Lamin C antibody [EP4520-16]	Abcam	Cat# ab215495, RRID:AB_3073679
Anti-mouse Pacific Blue(TM) Ly-6G/Ly-6C (Gr-1)	Biolegend	Cat# 108429 (also 108430), RRID:AB_893559
Alexa Fluor(R) 647 anti-Nuclear Pore Complex Proteins	Biolegend	Cat# 682203 (also 682204), RRID:AB_2728507
pS346/pS347-CXCR4 (phospho-CXC Chemokine Receptor 4 Antibody)	7TM Antibodies	Cat# 7TM0071B
CXCR4 (non-phospho), CXC Chemokine Receptor 4 Antibody	7TM Antibodies	Cat# 7TM0071N
Anti-G protein alpha Inhibitor 2 antibody [EPR9468]	Abcam	Cat# ab137050, RRID:AB_2773042
Anti-mouse Rab5 (C8B1)	Cell Signaling	Cat# 3547T,

		RRID:AB_2300649
Anti-mouse Rab7 (D95F2)	Cell Signaling	Cat# 9367T,<br>RRID:AB_1904103
Anti-mouse Rab11a	Cell Signaling	Cat# 2413S,<br>RRID:AB_2173452

Movie S1. CXCL12-induced intracellular Ca^{2+} flux in leukocytes. Live-cell imaging of free intracellular Ca^{2+} by Fluo-4 fluorescence (green) after stimulation of $CD45^{+}$ leukocytes (red) with CXCL12. The movie corresponds to fig. S1I.

Movie S2. High basal free Ca^{2+} and no CXCL12-induced rapid changes in free Ca^{2+} in erythroblasts. Live-cell imaging of free intracellular Ca^{2+} by Fluo-4 fluorescence (green) in $ACKR1^{+}$ nucleated erythroid cells (red) after stimulation with CXCL12. The movie corresponds to fig. S1I.

Movie S3. Endocytosis of CXCL12 and CXCR4 in erythroblasts. A 3D confocal microscopy image of a freshly isolated, viable erythroblast pre-stained with anti-Ter119 (blue) and anti-CXCR4 (green), followed by stimulation with CXCL12-AF647 (red). The image was captured 60 min after the addition of CXCL12. The movie corresponds to Fig. 1F.

Movie S4. Nuclear localization of CXCR4 and phosphorylated CXCR4 in an erythroblast. A 3D confocal microscopy image of a freshly isolated, viable erythroblast stimulated with CXCL12 and stained with anti-CXCR4 (green), anti-pS346 CXCR4 (red), anti-Ter119 (blue), and Hoechst (white). Scale bar, 5 μm. The movie corresponds to Fig. 4A.

Movie S5. CXCL12 stimulates erythroblast elongation, CXCR4 redistribution, nuclear polarization, and chromatin condensation. Live-cell imaging of freshly isolated, viable erythroblasts pre-labelled with anti-CXCR4 antibody (green), anti-Ter119 antibody (blue), and the nuclear dye Hoechst (white) and then stimulated with CXCL12-AF647 (red), which was added during imaging. The movie corresponds to fig. S8A.

Movie S6. CXCL12-stimulated erythroblast elongation, nuclear polarization, chromatin condensation, and rapid perinuclear Ca^{2+} transients. Live-cell imaging of freshly isolated, viable erythroblasts pre-labelled with the Ca^{2+}-sensitive dye Fluo-4 (green), anti-CD71 antibody (white), and the nuclear dye Hoechst (blue) and stimulated with CXCL12-AF647 (red), which was added during imaging. The movie corresponds to Fig. 5A.

Data file S1. Differentially expressed genes (DEGs) between CXCR4-deficient versus -sufficient cells; GO enrichment analysis of genes that were reduced in expression in CXCR4-deficient erythroblasts; GO enrichment analysis of genes that were increased in expression in CXCR4-deficient erythroblasts; upstream kinase analysis (UKA).
